# A generic approach to identify Transcription Factor-specific operator motifs; Inferences for LacI-family mediated regulation in *Lactobacillus plantarum *WCFS1

**DOI:** 10.1186/1471-2164-9-145

**Published:** 2008-03-27

**Authors:** Christof Francke, Robert Kerkhoven, Michiel Wels, Roland J Siezen

**Affiliations:** 1 TI Food and Nutrition, P.O. Box 557, 6700AN Wageningen, The Netherlands; 2 Center for Molecular and Biomolecular Informatics (260), NCMLS, Radboud University Nijmegen Medical Center, P.O. Box 9101, 6500HB Nijmegen, The Netherlands; 3 NIZO food research, P.O. Box 20, 6710BA Ede, The Netherlands

## Abstract

**Background:**

A key problem in the sequence-based reconstruction of regulatory networks in bacteria is the lack of specificity in operator predictions. The problem is especially prominent in the identification of transcription factor (TF) specific binding sites. More in particular, homologous TFs are abundant and, as they are structurally very similar, it proves difficult to distinguish the related operators by automated means. This also holds for the LacI-family, a family of TFs that is well-studied and has many members that fulfill crucial roles in the control of carbohydrate catabolism in bacteria including catabolite repression. To overcome the specificity problem, a comprehensive footprinting approach was formulated to identify TF-specific operator motifs and was applied to the LacI-family of TFs in the model gram positive organism, *Lactobacillus plantarum *WCFS1. The main premise behind the approach is that only orthologous sequences that share orthologous genomic context will share equivalent regulatory sites.

**Results:**

When the approach was applied to the 12 LacI-family TFs of the model species, a specific operator motif was identified for each of them. With the TF-specific operator motifs, potential binding sites were found on the genome and putative minimal regulons could be defined. Moreover, specific inducers could in most cases be linked to the TFs through phylogeny, thereby unveiling the biological role of these regulons. The operator predictions indicated that the LacI-family TFs can be separated into two subfamilies with clearly distinct operator motifs. They also established that the operator related to the 'global' regulator CcpA is not inherently distinct from that of other LacI-family members, only more degenerate. Analysis of the chromosomal position of the identified putative binding sites confirmed that the LacI-family TFs are mostly auto-regulatory and relate mainly to carbohydrate uptake and catabolism.

**Conclusion:**

Our approach to identify specific operator motifs for different TF-family members is specific and in essence generic. The data infer that, although the specific operator motifs can be used to identify minimal regulons, experimental knowledge on TF activity especially is essential to determine complete regulons as well as to estimate the overlap between TF affinities.

## Background

Numerous studies have been devoted to the identification of Transcription Factor (TF)-binding sites or other regulatory elements in bacterial genomes. So far, most large-scale approaches relied heavily on statistics and the input of known binding motifs [1-7]. Unfortunately, purely statistical approaches are seriously hampered by the trade-off that exists between a high true-positive rate and a low false-negative rate of the prediction. Nonetheless, both rates can be considerably improved by taking advantage of additional data [2,8] like, for instance, sequence data from related species [9-11], structural information [12] or transcriptome data [13,14]. Another way to enhance the accuracy is phylogenetic footprinting which takes both 'phylogeny' and 'synteny' into account[8,14-16].

We have recently developed a large-scale automated regulatory motif prediction method for prokaryotic genomes [17]. It was applied with success in the identification of a relatively large number of regulatory motifs in genomes of the *Firmicutes*, a phylum that comprises many well-studied families like the *Bacillaceae*, *Clostridiaceae*, *Lactobacillaceae*, *Staphylococcaceae *and *Streptococcaceae*. The identified motifs included several new motifs besides known ones. Nevertheless, in many cases the method appeared less suited to couple a specific TF or signal to the regulatory motif in a straightforward manner. For example, although the characteristic T-box motif was easily identified – the T-box is a regulatory element that responds to uncharged t-RNA [18] and is found in all *Firmicutes *– the amino acid specificity of that element was not retrieved for the individual instances automatically (Wels et al. unpublished results). Likewise, the 'CRE-like' motif that was retrieved is very similar to known operator motifs of various TFs of the LacI-family, suggesting that the recovered motif is not specific.

The LacI-family of TFs plays a crucial role in many bacterial species, and certainly in those of the phylum *Firmicutes*, as these TFs mediate preferences in the utilization of certain carbohydrates over others. The prioritization involves both repression (or activation) of catabolic genes (i) in the absence (or presence) of a related substrate and (ii) in the presence (or absence) of a preferred substrate [19-21]. The latter process is referred to as carbon catabolite repression (CCR) and its main mediator in Firmicutes species is CcpA [21-25]. CcpA operators were called CREs (CcpA-responsive elements [26]) and a CRE consensus motif was defined on basis of experiments in various *Firmicutes *species [21-23,25,27-30]. The consensus motif is very similar to, and sometimes coincides with, operators related to other TFs of the LacI-family [30-33]. Most family members, however, interact with only a few operators on the genome, like LacI of *Escherichia coli*, which represses specifically the *lac*-operon in the absence of lactose [34]. This raises the question how these bacteria coordinate 'local' (def: control of the expression of one or a few genes/operons) and 'global' (def: control of the expression of many genes/operons) regulatory effects using homologous TFs.

Thus, the lack in specificity of the current prediction methods is a key issue in case one wants to disentangle complex regulatory relationships, like between those of the TFs of the LacI-family and the operons involved in carbohydrate catabolism. Therefore, we have formulated a comprehensive sequence-based comparative approach for the prediction of TF-specific operators in bacteria. Specificity is ensured by building upon a proper phylogenetic classification of each family of TFs (whose members can for instance be found in reference databases [35-37]) and very strict criteria to define synteny.

The value of the approach was put to the test on the well-described LacI-family of TFs, and more specifically, to uncover the regulatory connections of the 12 LacI-family TFs in *L. plantarum *WCFS1. This species was chosen as a representative of the phylum Firmicutes, as it is an industrially and medically relevant model organism that is encountered in very different environmental niches, i.e. in association with plants, fermenting food and feed, and in the animal and human gastrointestinal tract [38,39]. The approach proved successful and each LacI-family TF of *L. plantarum *was linked to a putative operator motif and thereby to a putative regulon. In addition, several principles that should govern LacI-family TF mediated 'local' and 'global' transcription regulation could be inferred from the results. Ample experimental and structural information was used to evaluate and support the predictions and inferences.

## Results

### I) A comprehensive approach to identify TF-specific operators

It has been observed consistently that orthologous protein sequences [40] are very likely to have molecular properties that are alike [41]. Similarly, synteny – conserved gene order – was found to be a strong indicator of functional equivalency [42]. Thus, genes that are orthologous and share 'gene context' can be assumed to be functionally more equivalent than orthologous genes that are not syntenous. Based on this premise we formulated a generic phylogenetic footprinting [43]/shadowing [44] approach for the identification of TF-specific operator sequences in bacteria (description in Methods). High specificity in the motif prediction was achieved by properly classifying orthologous TFs into groups that share gene context to yield putative Groups of Orthologous Functional Equivalents or GOOFEs. To develop the approach, we chose the well-described LacI-family of transcriptional regulators (PFAM PF00356), limiting the analysis to Firmicutes and focusing specifically on the model organism *Lactobacillus plantarum *WCFS1, which has a high number of LacI-family TFs for which we have ample experimental and transcriptome data for validation.

#### Collect homologs: LacI-family TFs in the genomes of *L. plantarum *WCFS1 and other *Firmicutes*

**Figure 1:**
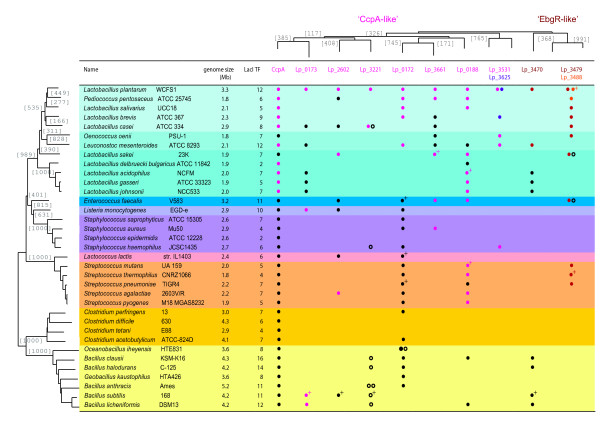
The number of LacI-family TF homologs and the presence of *Lactobacillus plantarum *orthologs in different *Firmicutes*. The organisms are organized on basis of their phylogeny (left; inferred from phosphoglycerate kinase amino acid sequence data [98]) and the TFs on basis of the NJ-tree of the *L. plantarum *LacI-family TFs (top). The presence of an ortholog to the *L. plantarum *proteins is indicated by open (different cluster in the NJ-tree) and closed circles (same cluster in the NJ-tree). The members of the various *L. plantarum *GOOFEs are colored. Some orthologs have been experimentally characterized and are indicated by '+'. **remark**: Although the PFAM HMM that is used to identify the LacI-domain represents only a small part of the DNA-binding domain, in most instances there was complete correspondence between the number of LacI-family TFs identified by us and the number listed by PFAM [96]. However, there were a few exceptions and in these cases the number given by PFAM appeared erroneous [see Additional file 1]. In some cases the PFAM database was just incomplete (e.g. *Pediococcus pentosaceus *and *Leuconostoc mesenteroides*). In other cases sequences were counted twice as a result of double Uniprot entries (e.g. for CcpA in *L. plantarum*). Other proteins were missing in the PFAM database because of mistakes in the ORF definition.

The search for LacI-family TF specific operators was initiated by collecting the LacI-family TF protein sequences from taxonomically related genomes and grouping them using the Neighbor Joining (NJ) algorithm (for relevant data see Additional files 1, 2, 3, 4). The resulting NJ-tree indicated a clear separation between two subfamilies of LacI-family TF homologs (top of Figure 1 and see Additional file 5). One subfamily represented the vast majority of LacI-family TF homologs including CcpA, whereas the other represented only 1 to 3 homologs per species. The latter subfamily contained one well-studied TF from *E. coli*, the evolved beta-galactosidase repressor or EbgR [45]. Henceforth, the two LacI-family TF subfamilies will be referred to as 'CcpA-like' and 'EbgR-like'. The number of LacI-family TF homologs ranged from 2 to 17 and the homolog composition was found highly variable between different species and also variable between strains (see Figure 1 and Additional file 1) and correlated roughly with genome size. For example, although all 6 LacI-family TFs of *Pediococcus pentosaceus *[46] were orthologous to a LacI-family TF in *L. plantarum *WCFS1, only 5 out of 9 where orthologous in *Lactobacillus brevis *[46], another close relative. Moreover, when the LacI-family TF content of various strains of *L. plantarum *was analyzed – the data were derived from a strain diversity analysis [47] – it appeared that apart from *ccpA *none of the individual LacI-family genes was present in all other strains. In fact, the master regulator CcpA was the only LacI-family member present in all sequenced *Firmicutes *genomes.

#### Determine synteny: Identification of TF-specific binding motifs

**Figure 2:**
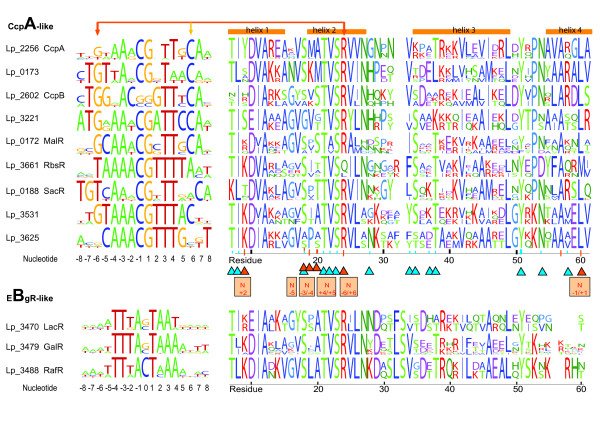
Left panel: Sequence motifs of predicted LacI-family TF specific operators in *L. plantarum*. Right panel: The protein sequence motif of the DNA-binding region of the LacI-family TFs per GOOFE. The numbering of the protein residues deviates slightly from that in the various crystal structures. This relates to the fact that the alignment includes some gaps that are necessary to accommodate all the LacI protein sequences that have been compared by us. The visualization of the sequences was created using Weblogo [99]. **remark**: NMR studies have shown that the hinge helix plays an important role in kinking the DNA whilst forming an alpha-helix (helix 4) and thereby stabilizing the induced fit of the recognition helix within the major groove of the operator [33,81]. In fact, the 3D-structures of operator-bound CcpA and LacI implicate many residues of helix 3 and 4 in the contact of the TF with the operator [56,57]. Moreover, the 3D-structures indicate that in both CcpA and LacI the same residues are involved. The DNA-protein contacts are indicated with triangles. The blue triangles mark the residues interacting with the phosphate backbone and the red triangles mark the residues interacting directly with a nucleotide (the position of the nucleotide is indicated in a box). In the case of Lp_0188 (SacR), a well-conserved guanine and corresponding cytidine are found at positions -7 and 7 of the operator, respectively. This suggests that the operator recognized by Lp_0188 (SacR) and its orthologs, is two nucleotides wider than that recognized by other 'CcpA-like' LacI-TFs. The 'EbgR-like' LacI-family TFs carry a conserved insertion before helix 3 and seem to lack the characteristic conserved alanine and leucine (or methionine in the case of RbsR) at position 60 of the hinge helix in the 'CcpA-like' LacI-family TFs. The absence of these residues coincides perfectly with the absence of the central CG nucleotide pair in the predicted Lp_3470 (LacR), Lp_3479 (GalR) and Lp_3488 (RafR) operators.

Inspection of the gene-neighborhood of the genes encoding LacI-family TF homologs in *L. plantarum *indicated that most of them are associated with genes encoding proteins that catabolize carbohydrates. Although the gene association appeared conserved in other genomes, it was mostly only true for a limited number of species. A TF-specific GOOFE was defined for each *L. plantarum *LacI-family TF on basis of context conservation and then the upstream regions preceding the conserved operons/genes were selected (see Methods for the precise procedure). Multiple sequence alignments, motif-finding methods, as well as visual inspection, were then used to identify potential GOOFE-specific LacI-family TF operator motifs for all 12 LacI-family TFs (see Additional file 6). A first comparison of the motifs, depicted in Figure 2, showed that the 'CcpA-like' and 'EbgR-like' LacI-family TF operators had characteristic yet distinct subfamily traits. The 'CcpA-like' operators carry a central CG nucleotide pair, whereas the 'EbgR-like' operators have only a single central C or G nucleotide. Moreover, within the subfamilies, the motifs appeared to be discretely different in at least one position.

#### Validation: Comparison of the predicted motifs with experimental functional data from literature

Compelling evidence that the recovered motifs were genuine and the approach was effective came from a comparison of the motif predictions with experimentally characterized operators. In all cases that could be checked, the prediction was in full agreement with the experimental findings. This was true for CcpA in *Lactobacilli *[48,49], for the Lp_3470 ortholog LacR in *Lactobacillus delbrueckii *subsp. *lactis *[50], for the Lp_3479 ortholog GalR in *Streptococcus thermophilus *[51] and in *Streptococcus mutans *[52], as well as for MalR in *Stretococcus pneumoniae *[53], for MalI in *E. coli *[31] and for ExuR in *Bacillus subtilis *[54] (see Tables 1, 2 and 3).

**Table 1:** The CRE consensus. For *B. subtilus *and species of the phylum *Firmicutes *in general, a consensus has been formulated by others on basis of both (exp) experiment and (pred) predictions. For the composition of the *L. plantarum *CRE consensus (bold, italics) we have used the two experimentally established CREs in *L. plantarum *[49,110] and the initial CcpA operator motif retrieved by us (Figure 2).

LacI-family TF	Organism	Site	Operator^(a)^	Evidence
CcpA	*B. subtilis*	CRE	TG WNAN CG NTNW CA	pred/exp: [29]
	*B. subtilis*	CRE	TG NAAR CG NWWW CA	pred/exp: [22,28]
	*L. lactis*	CRE	WG WAAR CG YTWW MA	pred/exp: [25]
	*Firmicutes*	CRE	WG NAAS CG NWWN CA	pred/exp: [30]
	*Firmicutes*	CRE	WG HWAD SG YWWD CA	pred/exp: [21] ^(b)^
	***L. plantarum***	**CRE**	***NK NWAN SG NWWN CA***	**pred/exp: [49, 110] and this work**

(a) Abreviations for specific nucleotide combinations taken from [111]: C, G: S (strong); A, T: W (weak); A, G: R (purine); T, C: Y (pyrimidine); T, G: K (keto); A, C: M (amino); A, T, G: D (not-C); A, T, C: H (not-G); A, T, C, G: N (any).

(b) This consensus is based on the experimentally verified operators listed in this reference

**Table 2:** Known and predicted operators for ExuR in *B. subtilis *and MalI in *E. coli*. The operators determined by experiment are shown in normal print and the same operators as predicted using our new approach are shown in bold *italics*. O_1 _and O_2 _indicate the relative position of the operator sequences with respect to the translation start.

LacI-family TF	Organism	Site	Operator	Evidence
ExuR	*B. subtilis*		TG TTAA CG TTAA CA	pred/exp: [54]
**ExuR**	***B. subtilis***		***TG TTAA CG TTAA CA***	**pred, this work**
MalI	*E. coli*	O_1_	GT AAAA CG TTTT AT	pred/exp: [31]
		O_2_	GA AAAA CG TTTT AT	
**MalI**	***E. coli***		***gT aAAA CG TTTT At***	**pred, this work**

**Table 3:** Operators for various LacI-family TFs present in *L. plantarum*. The operators that were verified by experiment in several species of the phylum *Firmicutes *are listed in normal print, the operators predicted by us for the orthologous TFs in *L. plantarum *are in bold *italics*. O_1 _and O_2 _indicate the relative position of the operator sequences with respect to the translation start. * Transcription from O_1 _was 10 times stronger than from O_2_.

CcpA-like LacI-family TF	Organism	Site	Operator	Evidence^(a)^
MalR	*S. pneumoniae*	O_1_	CG CAAA CG TTTT CC	pred/exp: [53]
		O_m_	CG CAAA CG TTTG CG	pred/exp: [53]
**Lp_0172**	***L. plantarum***		***cG CAAa CG cTTG CA***	**pred, this work**
RbsR	*L. sakei*		gT AAAA CG TTTT Ac	pred: [112]
	*E. faecalis*		gT AAAA CG TTTT Ac	
**Lp_3661**	***L. plantarum***		***.T AAAA CG TTTT Aa***	**pred, this work**

EbgR-like LacI-family TF				

LacR	*L. delbrueckii*	O1	TTG TTT ACT AAA AAT	pred/exp: [50]
		O2	TTG TTT AGT AAA CGG	pred/exp: [50]
**Lp_3470**	***L. plantarum***		***aaa TTT AGT AAT t..***	**pred, this work**
GalR	*S. thermophilus*		..T TTT AGT AAA A..	pred/exp: [51]
GalR	*S. mutans*	O1*	AAA TTT AGT AAA ATT	pred/exp: [52]
		O2*	ATT TTT ACT AAA ATT	pred/exp: [52]
**Lp_3479**	***L. plantarum***		***aat TTT AGT AAA a..***	**pred, this work**

#### Validation: Comparison of the predicted with 3-D structure information from literature

It also proved possible to use structural information on the binding of several LacI-family members to their respective operator [55-57] to validate predicted motifs. Differences in the conservation of certain amino acid residues in the DNA-binding domain of the TF were compared to the composition of the connected operator. Two clear correlations between protein sequence and operator sequence were found (see also the legend to Figure 2):

- Firstly, the structural data suggest that, in the case of CcpA and LacI, the conserved arginine located at position 24 is one of the few residues that hydrogen bonds directly with one of the nucleotide bases, a guanine at position -6 of the operator [56,57]. In Lp_3661 (RbsR) and its orthologs, the arginine is replaced by a glutamine (or leucine) and correspondingly the otherwise 'conserved' guanine is replaced by a thymidine. In fact, such a replacement was observed for all other studied LacI-family TFs deviant at position 24 (see Additional file 7). These anomalous TFs include MalI from *E. coli *which was proven experimentally to indeed bind an operator with a thymidine at position -6 [31] (Table 2).

- Secondly, the 'EbgR-like' TFs (i.e. Lp_3470 (LacR), Lp_3479 (GalR) and Lp_3488 (RafR)) are expected to have distinct DNA-binding features. Members of this subfamily lack the conserved leucine residue (position 60 in Figure 2) that according to the 3D-structure of operator-bound CcpA [57] intercalates between the central CG base pairs that are characteristic for 'CcpA-like' operators [22]. Concordantly, the predicted 'EbgR-like' LacI-family TF operators lack the central CG nucleotide pair. Possibly, the conserved arginine at position 24 interacts with the conserved single C or G nucleotide in the operator (Table 3).

### II) Identification of the biological role of a TF through comparative genomics

The biological role of a transcription factor is to activate or repress the transcription of certain genes in response to the presence of a signal (e.g. a nutrient or metabolite). In principle, once the sequence of a TF-specific operator is known, a genome-wide search for the related motif could be used to find putative TF-binding sites on the genome and to establish the regulated functionalities (regulon). The signal that triggers the transcriptional response can be obtained by linking the specific TF to an ortholog that has experimentally verified 'inducer' specificities. Finally, the transcriptional effect (i.e. activation or repression) of the binding of the TF can be deduced from the relative position of the putative binding site with respect to the promoter [25,58].

#### Regulon predictions for the LacI-family TF homologs in *L. plantarum *WCFS1

**Figure 3:**
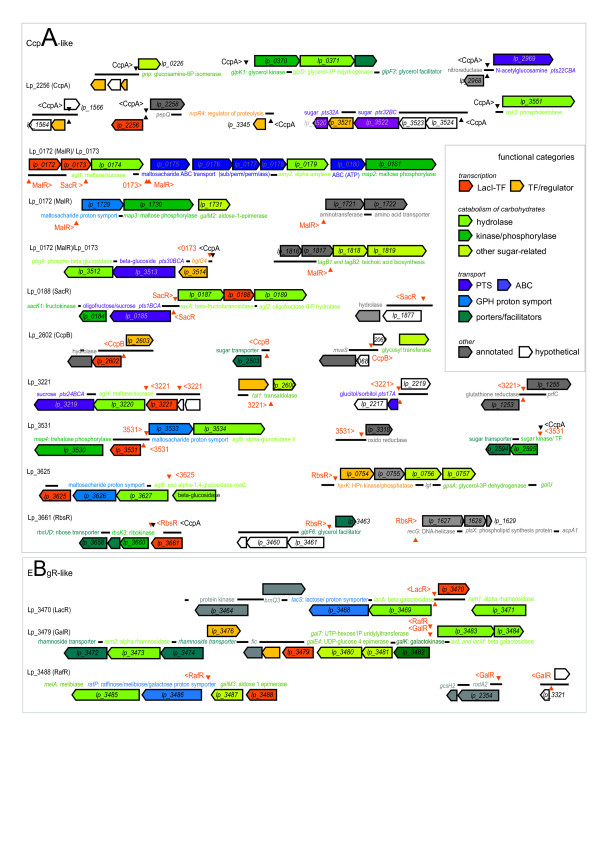
The *L. plantarum *operons predicted to be controlled by **(a) **'CcpA-like' LacI-family TFs and **(b) **'EbgR-like' LacI-family TFs. The set of operons is restricted to those having a very high probability of being correctly predicted. The positions of putative operators are marked by triangles and the direction in which transcription is presumably regulated is indicated (< and >). The functional categories of the proteins encoded by the genes that are under the control of LacI-family TFs are color-coded as depicted in the inset. The functional annotations were taken from the in-house annotation database of *L. plantarum *WCFS1 ([38] and C. Francke unpublished results). See [Additional file 9] for a detailed description of the functional annotation.

The 12 predicted specific operator motifs were used to search the genome for potential TF-binding sites. For each of the identified specific motifs an initial list of 30 to 100 putative binding sites was retrieved and the list was reduced by application of a distance and similarity criterion to yield a few putative highly specific binding sites per TF (visualized in Figure 3; data in Additional file 8). Not surprisingly, the 'best hits' included those sites that were used to create the search motif in the first place. However, they also included multiple sites that were not used as input. For instance in *L. plantarum*, new Lp_0172 (MalR) operators were detected upstream of the operon comprising the gene *lp_0172 *(*malR*) and upstream of the neighboring operon. Furthermore, the notion that auto-regulation is an important feature connected to LacI-family TF mediated regulation [27,49-51,54] was confirmed within *L. plantarum*, by the identification of a specific binding site upstream of all LacI-family TFs with the exception of Lp_0173, Lp_3488 (RafR) and Lp_3661 (RbsR). It is generally accepted that auto-regulation provides stability to a transcriptional network [59-61].

As expected, most potential binding sites were identified upstream of operons that encoded functionalities related to the catabolism of particular carbohydrates. In *L. plantarum *WCFS1, 11 out of 12 LacI-family TFs were found to be associated with active carbohydrate transport systems (driven by protons: GPH family; ATP: ABC transport systems; or phosphoenolpyruvate: PhosphoTransferaseSystems). Furthermore, the size of the putative regulons varied slightly. For instance, the putative regulon of Lp_3625 encompassed only one operon, whereas that of Lp_0172 encompassed five operons (Figure 3). Although the putative regulon of CcpA was the largest, it was still limited in size, which is slightly in contrast with the global role of CcpA [21,24,25]. The precise composition and functionality of most of the predicted regulons is discussed in some detail in Additional file 9.

#### The molecular function of LacI-family TFs and the connection with the predicted biological role

**Figure 4:**
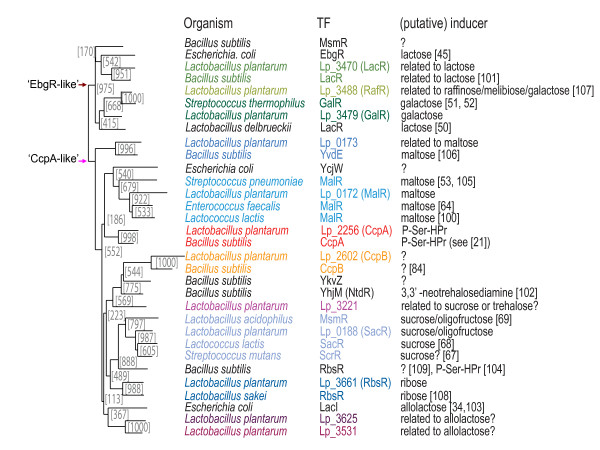
A reduced NJ-tree of the inducer-binding domain for the LacI-family TF homologs of *L. plantarum*. The sequences of LacI-family TF homologs with known inducer from other organisms were added for comparison [21, 34, 45, 50-53, 64, 67-69, 84, 100-109]. Orthology is indicated by color-coding. The numbers accompanying the clusters in the NJ-tree represent the bootstrap support for the individual divisions (out of 1000).

The functional similarity between homologs can be derived from a proper phylogeny of all homologous sequences [41,62]. However, the low bootstrap support for the 'early' branches in the NJ-tree of all LacI homologs made it impossible to deduce functional similarities between the members of different clusters of orthologous sequences (Methods and see Additional file 4). It was observed by us (Francke et al. unpublished results) and others [63] that generating a NJ-tree of intra-species homologs of specific functional domains is extremely helpful to overcome this problem. To obtain putative links with experimental functional data, orthologous sequences linked with experimentally verified 'inducer' specificities can be added. The branching pattern within such a NJ-tree for the LacI-family TFs of *L. plantarum *(Figure 4 and see Additional file 5) and the bootstrap support for that pattern, suggested clear similarities in the encoded affinity for certain inducer substrates. It must be noted here that 'inducer' does not necessarily mean that the binding of the TF to the DNA is promoted by the particular molecule. In fact, in many cases the interaction with the 'inducer' causes a release of the LacI-family TF from the DNA and thereby a relief from repression (as shown experimentally for MalR of *E. faecalis *[64]; SacR in *L. plantarum *[65]; GalR in *S. thermophilus *[51]; and RbsR in *L. sakei *[66]).

The identification of various additional sites whose presence should be expected (i.e. related to autoregulation or regulation of genomically associated operons) supported the view that the approach yielded genuine TF-specific binding sites. Other support for the validity of the identified sites and regulons was provided by a comparison of the functionalities encoded by the regulons and the molecules that induced the activity (or better: the in-activity) of the related TFs. Figures 3 and 4 show that in almost all cases a straightforward metabolic link existed between the predicted regulated functionality and the assigned 'inducer' of the TF. For example in *L. plantarum*, Lp_0188 (SacR) is predicted to respond to sucrose or oligofructose, a prediction that was derived from experimental evidence obtained for orthologous TFs [67-69]. Concordantly, its putative operators are found upstream of two operons that harbor the genes encoding an active oligofructose/sucrose uptake system [65] and enzymes that catalyze the conversion of the phosphorylated oligosaccharide into phosphorylated disaccharide and the phosphorylated disaccharide into glucose-6-P and fructose.

Some of the predicted regulatory connections could be substantiated directly by published transcription data for *L. plantarum *or related species. On the other hand, the predictions also could often not be extrapolated in a straightforward way. For instance, similar to the prediction for *L. plantarum*, the expression of the ribose utilization operon (*rbsUDK*) in *L. sakei *was shown to be controlled by RbsR and induced by ribose [66]. Unfortunately, the induction of other operons was not studied. Another example is provided by transcriptome data for *L. plantarum *grown on short-chain fructooligosaccharides compared to glucose. As predicted, expression of the divergon associated with *lp_0188 *was induced under these conditions [65]. Nevertheless, the maltase/sucrase encoding gene *lp_0174 *that was predicted to be controlled by Lp_0188 (SacR) was not induced. This observation could very well relate to additional factors that are involved in the regulation of the particular gene. An example of the subtle differences between species is found for the regulation of the *gal *operon (*galK*, *galT *and *galE*) and *lac *operon (*lacS *and *lacZ*). In *S. mutans *[52], *S. thermophilus *CNRZ 302 [51] and *S. salivarius *[70] expression of the *gal *operon, as well as that of *galM *and the *lac *operon in *S. thermophilus *and *S. salivarius *was shown to be controlled by GalR and induced by galactose. Our predictions suggest that in *L. plantarum *the *gal *operon is similarly controlled by the GalR ortholog Lp_3479. The *lac*-operon in *L. plantarum *however, was predicted to be controlled by a paralogous LacI-family TF, Lp_3470 (LacR), that is absent from the *Streptococci*, but which is present in *L. acidophilus *where it was shown to regulate an integrated *lac*-*gal *operon [71]. At the same time, in some strains of another Lactobacillus species (*L. delbrueckii *[50]) the *lac*-operon was shown again to be controlled by an ortholog of Lp_3479 (GalR).

#### The mode of action: repression or activation

**Figure 5:**
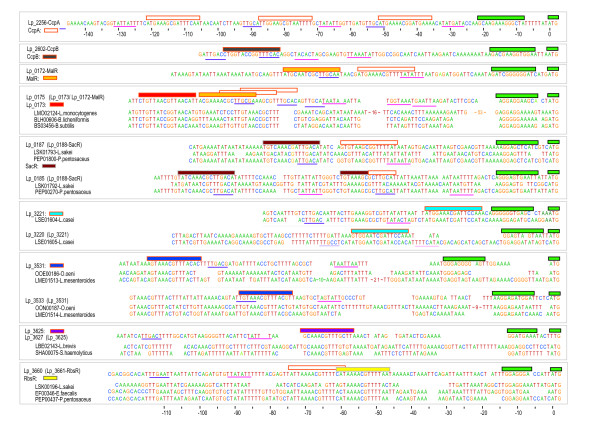
Operators present in the neighborhood of the genes encoding the LacI-family TFs of the 'CcpA-like' subfamily. For most TFs an alignment of the upstream region is shown for the sequences related to one GOOFE. In the case of CcpA, CcpB and Lp_0172 (MalR), no proper alignment could be made with regions from other organisms. Potential CREs are indicated by orange bordered boxes and the LacI-family TF specific operators are indicated by differently colored boxes. The -35/-10 regions of the putative promoters are underlined in purple and pink, respectively. The translation start is positioned at the right end and is indicated in green, as is the putative ribosome binding site.

Although, the nomenclature of most LacI-family TFs hints that their main mode of operation is repression (hence: Repressor), for CcpA it has been shown that it can also act as activator [21,23]. In *Lactococcus lactis*, activation by CcpA was observed when the central nucleotide of the CRE was located at position -31 or -21 with respect to the -35-sequence of the promoter and repression by CcpA, when it was located around positions -9, -4, +9, +19, +40, +50 and further downstream [25]. The characteristic intervals of 5 or 10.5 bases were ascribed to a helix-face dependence of the regulatory activity. A similar dependence had been observed before in the activation of *ackA *transcription in *B. subtilis *[72]. The footprints accumulated in Figure 5, indicated that most LacI-family TFs in *L. plantarum *are indeed expected to act as repressor. Moreover, in all cases the predicted operators are found at the expected characteristic positions with respect to the promoter.

#### Regulatory overlap between LacI-TFs

Another interesting aspect of the predicted regulatory connections that became apparent from inspection of the footprints in Figure 5 was that many operons appear to be preceded by multiple LacI-specific putative operators. For example, the two neighboring operons involved in sucrose/oligofructose transport and catabolism are preceded by two putative Lp_0188 (SacR) operators. It was shown that transcription of these operons is indeed induced simultaneously [65]. This observation fits the assumption that one of the operators controls the transcription in one direction and the other in the opposite direction. Conversely, two divergently transcribed genes can in principle also be controlled by a single operator. The latter was shown to be the case in the transcriptional control of the gene *levR *and the operon *levABCDX *in *Lactobacillus casei *[73] and in the transcriptional control of the genes *pepQ *and *ccpA *in *L. delbrueckii *[74] and *L. lactis *[25]. The genes *pepQ *and *ccpA *are similarly organized in *L. plantarum*. Furthermore, upstream of the *ccpA *gene three different putative promoter sites can be distinguished and every promoter seems to be connected to its own CcpA operator. This finding is in line with the experimental evidence provided by [49]. Nevertheless, based on the relative positions of the putative CREs, the effect of CcpA on its own expression is extremely difficult to predict of hand. CcpA seems to act as activator as well as repressor depending on the actual promoter.

#### The role of TF concentration

**Figure 6:**
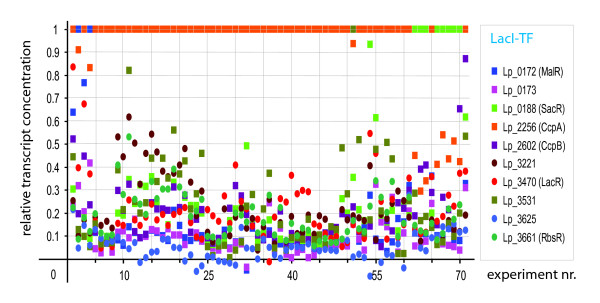
The relative absolute levels of LacI-family TF related mRNAs in various microarray experiments with *L. plantarum*. The related array data were used before by [17]. Information on the determination of these levels can be found in the Materials and methods.

The molecular nature of the interaction between TF and operator dictates that the actual binding of the two will be dependent upon the activity (in the thermodynamic sense) of both. Consequently, the occupancy of any binding site by a certain TF can be raised by raising TF concentration. In fact, a relatively high TF concentration is anticipated for CcpA [21]. To get some idea of the relative concentrations of the LacI-family TFs in *L. plantarum*, transcript levels obtained under different growth conditions were inspected (see Figure 6). The observed transcript levels suggested that except for Lp_3625, all LacI-family TFs are under some conditions expressed to relatively intermediate levels and Lp_0172 (MalR), Lp_0188 (SacR), Lp_3531, and CcpB even to levels as high as, or even higher than CcpA.

## Discussion

### A generic method to identify TF-specific operators

Every line of evidence sustains the validity of the approach we have formulated to identify LacI-family TF specific operator motifs. In all cases where a LacI-family TF operator has been characterized experimentally, our prediction is in full agreement (see Tables 1, 2 and 3). And likewise, several correlations between the TF sequence (protein) and operator sequence (DNA) that are anticipated on basis of structural information [56,57] were retrieved perfectly. Moreover, the fact that a specific operator motif could be identified for every LacI-family TF and that relatively few proper hits for these operator motifs were found on the complete genome, is proof by itself. More non-specific methods inevitably would have yielded more degenerate motifs and more false-positive identifications.

In lactic acid bacteria many operons involved in carbohydrate catabolism are associated on the genome by the gene encoding the respective regulator [75]. In fact, this observation may be generalized for all TFs that are considered 'local' regulators. Our results indicate that especially for these TFs, a distribution into Groups of Orthologous Functional Equivalents will reduce the noise in the motif prediction significantly. In contrast, as current automated methods generate more degenerate motifs [17] these methods are better suited for the recovery of binding sites for 'global' regulators.

### Characteristic motifs and the implications of degeneracy

As the interaction between TF and DNA allows for a certain structural freedom, a TF-specific operator is not necessarily a unique sequence but merely a collection of sequences which can be represented as a motif or consensus sequence. The molecular nature of the interactions dictates a distinct relationship between the affinity of the TF protein for the operator DNA and their respective sequence. As a consequence, a more degenerate operator motif relates to reduced affinity. For example, the LacI-family TF CytR in *E. coli *exhibits a more versatile binding of operator sequences than LacI (i.e. has a higher motif degeneracy). At the same time it was observed that its affinity for the operator is much reduced when compared to LacI [76,77]. Likewise, the affinity of the TF for the operator was shown to be affected significantly by subtle changes in the protein sequence [78] as well as in the nucleotide sequence of the operator (for LacI: [79-81]; for CcpA: [29]). Lehming et al. therefore [78] assumed explicitly that the interaction between TF and operator should be concentration dependent. Ultimately, it is the relation between the concentration (or better: activity) of active TF and the rate of expression that determines key features of the dynamics of the cellular response to internal and external signals [82].

The predicted specific operator motifs of the LacI-family TFs in *L. plantarum *exhibit relatively little degeneracy (>8 nucleotides fully conserved for the 'CcpA-like' subfamily; see Figure 2) with one exception: the operator motif of CcpA itself. Considering the above, and based on the fact that in the 3D-structures of CcpA and LacI bound to their respective operators the same residues are involved in the interaction of TF with DNA [56,57], the degeneracy of the CcpA operator motif indicates it should act at relatively higher concentrations with respect to LacI and other relatives. Concomitantly, variable regulation of *ccpA *expression would represent a way to control the differential binding of CcpA to CREs [21].

### 'Local' versus 'global' regulation

The identified CcpA operator motif (CRE) of *L. plantarum *is very similar to the consensus CRE that was initially defined for *B. subtilis *on basis of a site-directed mutagenesis study [29] and later refined on basis of the experimental identification of additional CREs [22,30] (CRE consensus sequences are summarized in Table 1). Remarkably, the DNA-binding domain of CcpA on the protein level is considerably more conserved compared to that of the other LacI-family TFs (see Figure 2 right panel), whereas in contrast, the operator motif is the most degenerate. Both facts reflect and emphasize the 'global' role of CcpA. We observed that the CcpA regulon that was defined on basis of a genome wide search with the specific operator motif was relatively small. The same observation was made by [22] when the genome of *B. subtilis *was searched for potential CREs for the first time. The authors concluded that this related to the lack of degeneracy in the search motif and they proved experimentally that this was indeed the case.

It is generally assumed that transcription and translation are connected processes in bacteria [83] and as a consequence proteins should be produced in the physical vicinity of where they are encoded. A major implication of an intended local role of a TF would then be that the number of TF molecules necessary to effectively control expression can be minimized in case the affinity for the operator is relatively high (signified by a less degenerate motif). As mentioned in the previous section, all but one of the predicted operators indeed show a relatively high degree of conservation over different, sometimes even distantly related, species. A low TF concentration will keep in check non-local interactions as the TF will be virtually absent in the rest of the cell and, as a result, even operators that are very similar will not be affected. In fact, it was shown for carbohydrate utilization by *Lactobacillus acidophilus *that induction of catabolic operons is highly specific for distinct sugars [71]. Vice versa, a higher TF concentration, like anticipated for CcpA [21], would relax the sensitivity towards the composition of the operator and thus enable binding to sites for which the TF has less affinity. However, transcript levels that are observed in *L. plantarum *under different growth conditions are not completely conclusive (see Figure 6). Nevertheless, based on the observed transcript levels one should expect that Lp_0172 (MalR), Lp_0188 (SacR), Lp_3221, Lp_3531, Lp_3661 (RbsR), CcpB and CcpA in principle could regulate multiple and also distant operons.

### Regulon boundaries and induced response

Searching the genome of *L. plantarum *with the identified specific operator motifs yielded a list of potential binding-sites for every LacI-family TF. To avoid many false predictions, we have used two conservative criteria to reduce the list of putative TF-specific binding sites. They related to the position of the site with respect to the translation start, as there is experimental data showing certain boundaries for that distance [21,23], and to a maximum number of 2 deviating nucleotides. The genes/operons preceded by the putative binding sites thus should constitute putative minimal regulons. In principle, more degenerate motifs should lead to a longer list of compliant sites, as was indeed observed. This observation, which was earlier made by others [22], reveals a key point in regulon predictions based on operator motifs, namely motif degeneracy complicates a straightforward decision about the authenticity of the recovered sites. Moreover, as described in the above sections, binding will by necessity be influenced by TF concentration (activity). Therefore, experimental data on gene expression *and *TF concentration (activity) will be essential to refine the predictions. At the same time, in most cases, a proper interpretation of experimental transcription data will require motif and regulon predictions because of the fact that the activity of many TFs is intertwined and the number of conditions tested or testable too limited to untwine these. Although the extrapolation of the predictions to experimental data is non-trivial, several of the predicted associations could be confirmed on basis of data obtained in *L. plantarum *and related species (see Results). Moreover, a comparison of the predicted regulons depicted in Figure 3 with the environmental signals that are expected to govern the specific LacI-family TF activities (see Figure 4) shows that the recovered connection make perfect biological sense. This finding strongly supports the assertion that the predictions provide a valid coupling between the LacI-family TFs and functionalities encoded by the putative regulons.

## Conclusion

We have formulated a sequence-based approach that enables the identification of TF-specific binding motifs. One of the major advantages of the approach is that it is generic and thus, in principle, can be applied to any TF family without prior knowledge of the actual composition of the binding motif. In fact, we are in the process of performing similar analyses for various TF-families, including two component systems, and the preliminary results confirm the assertion. The method appears perfectly suited to identify binding sites on the genome connected to local regulators in contrast to current automated procedures that yield mostly sites connected to global regulators.

The presented data substantiate the successful identification of specific operator motifs related to the LacI-family TFs in the model organism *L. plantarum*. The recovered motifs differ in at least one position but at the same time their similarity is considerable. As the composition of the operator motif is tightly related to the affinity of the TF for the DNA this finding implicates that some of the LacI-family TFs could potentially bind to the operators of another. In fact, the observed competition in *B. subtilis *TF knock outs, between CcpA and CcpB in the repression of the *gnt *and *xyl *operon [84], exemplifies this phenomenon. Simultaneously, higher TF (or binding site) concentration (activity) will result in regulation at degenerate sites (i.e. lower affinity) (see [1]), a conclusion that correlates well with the mechanism of control of TF-activity itself as this involves a change in affinity of the TF for the operator upon induction [34,85]. An important corollary is that regulons, and especially those related to global regulators, will vary in size depending on the environmental conditions.

Finally, potential binding sites can be identified based on the operator motif predictions and from those the functionalities that are regulated in response to a given stimulus can be reconstructed. In principle, the coupling of putative regulons with potential TF inducers thus provides insight in the prioritization of the functionalities within a certain organism. Nevertheless, our data on LacI-family TFs in *L. plantarum *makes perfectly clear that in order to arrive at a complete reconstruction of the encoded transcriptional response to environmental stimuli, experimental data on transcription as well as TF and inducer concentration under different environmental conditions is adamant.

## Methods

### Resources and tools

All genomic information was obtained from the ERGO genome analysis and discovery system [86] and updated until the 1st of July 2007. Nevertheless, the presented results do not depend on the use of this particular resource and the methods described in this paper can as well be applied using publicly accessible resources (like those at NCBI [87]). The genome sequence of *L. plantarum *WCFS1 and the functional annotation of its genes was taken from our in-house annotation database [38]. Potentially homologous sequences were collected from the database using the BLAST algorithm [88], with a typical cut-off between 10^-2 ^and 10^-10^. Multiple sequence alignments were made with MUSCLE [89] (default settings). Alignments were visually inspected and aberrant sequences were removed (characterized by many gaps and a distinctly different conservation pattern). BioEdit [90] and Jalview [91] were used to edit sequences, and ClustalW [92] was used to create (domain-) specific bootstrapped neighbor-joining trees (with 'correction for multiple substitutions' [93]). The resulting trees were analyzed using LOFT, a tool that automatically divides the sequences into orthologous groups based on the hierarchy of the tree and the duplication and speciation events implied by that hierarchy [62]. Overrepresented DNA sequences in a selected set of upstream regions (300 bases) were identified automatically using MEME [94] and MAST [95] was used to detect other potential TF-binding sites on the genome (default cut-off p-value < 10^-5^).

### Identification of TF-specific operator motifs

**Figure 7:**
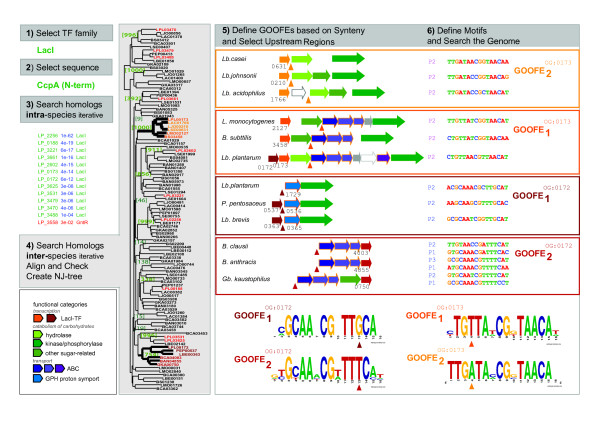
The TF-specific operator motif identification workflow. 1) First a particular TF-family was selected and 2) a prominent representative of that family was chosen. 3) The related sequence was used to search the genome of a particular species for intra-species homologs. This search was iterated until no new sequences are recovered. A high e-value cut-off was employed to ensure the recovery of all homologs. The sequences were aligned and a NJ-tree was generated. Both the alignment and the NJ-tree were used to determine the family or sub-family boundaries. 4) The procedure was repeated to retrieve all inter-species homologs and the general features of the intra-species homologs were used to determine the sequences that were taken into consideration. Orthologous relations between sequences were established on basis of clustering in the NJ-tree and a sufficient bootstrap support (in green) for the clustering. In the case of Lp_0172 and Lp_0173 the orthologous clusters are color-coded in brown and orange, respectively, and the other TFs of *L. plantarum *are indicated in red. 5) The genomic context of the various orthologs was inspected (legend bottom left) and in case clear differences existed, the orthologous groups were sub-divided into different Groups of orthologous functional equivalents (GOOFEs), as illustrated. Then, upstream regions of the conserved gene(s) in context were selected and inspected for potential regulatory sequences (the selected regions are indicated by colored triangles). The potential regulatory sequences were compared and those that showed similar features were selected. In fact, only those sequences that showed the highest conservation were selected to determine a specific operator motif. In the case of Lp_0172 and Lp_0173, a 'CcpA-like' operator motif was found up to 3 times in the upstream regions. The sequences that were selected to determine the Lp_0172 and Lp_0173 specific operator motifs are displayed (Px indicates the relative position of the selected sequence with respect to other similar sequences and relative to the translation start). 6) The selected sequences were used to create a GOOFE specific operator motif. The thus identified specific motifs related to the orthologous groups containing Lp_0172 and Lp_0173 demonstrate that the division into GOOFEs was essential to arrive at highly specific operator motifs. Although the motifs within both orthologous groups are highly similar, they differ distinctly in one position depending on the GOOFE. In the case of the TFs orthologous to Lp_0172, the motifs are strikingly different at position +5, with a fully conserved guanine in the GOOFE containing Lp_0172 and a fully conserved thymidine in the other. And in the case of the TFs orthologous to Lp_0173 the motifs are strikingly different at position -5, with a fully conserved thymidine in the GOOFE containing Lp_0173 and a fully conserved adenine in the other. **remark**: The gene/protein identifiers in the figure are derived from the ERGO resource [86]. A conversion to other identifiers can be found in [Additional file 2]. The functional annotation of the depicted genes were taken from the in-house annotation database of *L. plantarum *WCFS1 ([38] and C. Francke unpublished results) and the ERGO resource. See [Additional file 9] for a detailed description of the functional annotation in *L. plantarum*.

A generic phylogenetic footprinting/shadowing approach was formulated to improve the identification of TF-specific operator motifs. Compared to other methods the specificity of the motif prediction is increased by the identification of orthologs *proper *and by taking into account the modular organization of the bacterial genome. The approach was applied to a model family of TFs (LacI) in the model organism *L. plantarum *WCFS1. The related flow scheme is depicted in Figure 7 and described in detail below:

#### - Selection of a TF family, the collection of homologs and the derivation of orthology (Figure 7 1–4)

Intra-species and inter-species homologs were collected from the database using BLAST and the search was iterated until no additional sequences were found. This search was not only performed on the level of the complete sequence but also with individual functional domains. The sequences were aligned, aberrant sequences were removed, a bootstrapped NJ-tree was generated, and the hierarchy of the branching together with the bootstrap support were considered to identify orthologs. In the case of LacI-family members, the complete sequence of CcpA from *L. plantarum *was used as a starting sequence, as well as the N-terminal (first 90 residues; DNA binding domain) and C-terminal (other residues; inducer binding domain) sequence. To restrict the size of the final collection, only *Firmicutes *genomes were analyzed. The examined species included well-studied organisms such as *B. subtilis*, *L. lactis *and *S. thermophilus *(see Additional file 1 for a complete list of analyzed genomes). To improve the potential for functional identification the genome sequences of several *E. coli *strains and *Salmonella *species were also included. A striking feature of the NJ tree of the *Firmicutes *LacI-family TF homologs was that the representation of the 'early' branching events came out very unreliable, as signified by the extremely low bootstrap support (several values were as low as 1). In contrast, most branches related to supposed more recent evolutionary events had high bootstrap values in the NJ-tree and, as a result, the LacI-family TF homologs could be separated reliably into groups of orthologous sequences (see Additional files 4 and 5). The set of homologs identified by us was compared to the entries in the PFAM database [96].

#### - Definition of functional equivalents (Figure 7 4,5)

Orthologous clusters can often be further subdivided to obtain putative Groups of Orthologous Functional Equivalent s or GOOFEs. The homogeneity of the sequence alignment (as indicated by conserved stretches of residues and the absence of large gaps or inserts), a high bootstrap-value at the branching point that separates the orthologous cluster from the other sequences (Figure 7 4), and most importantly, a clear difference in conserved gene-context within the group were used to evaluate the necessity of such sub-division (Figure 7 5). In the case of many of the LacI-family TFs of *L. plantarum*, the subdivision into GOOFEs resulted in clearly distinct operator motifs even within an orthologous group (as illustrated in Figure 7). The protein sequences, alignments and trees can be found in Additional files 1, 2, 3, 4, 5.

#### - Selection of upstream regions containing putative operators (Figure 7 5)

The observation that most genes encoding TFs seem to be associated on the genome with the genes whose transcription they control may guide the selection of upstream regions. The upstream regions of the conserved operons within a GOOFE were used to search putative operator sites (selected regions (see Additional file 6)). Only, in case the TF encoding gene lay solitary on the genome the upstream regions of the TFs from one GOOFE were used, based on the notion that autoregulation is a common feature of many TFs.

#### - Motif definition (Figure 7 6)

Potential TF binding regions on the DNA (*i.e. *operators) were searched automatically in the selected set of upstream regions (300 bases) using MEME. As motif prediction tools often produce multiple motifs including many false positives, an alignment of the regions was made and the observed conservations were compared to the automatically recovered motifs to remove most false positives. The final collection of motifs was then compared within the complete TF-family and the TF-specific motifs were defined based on conserved features, like characteristic residues, spacing and motif length. The LacI-family TFs are known to form functional dimers and as a consequence the reported binding sequence motifs for these proteins are palindromes of lengths varying between 10 and 16 basepairs [55,57,81]. Therefore MEME was tuned to find inverted repeats (-pal option) with a maximum width of 20 bases and the detection of 4 different motifs with zero or one occurrence per sequence (-ZOOPS option). The resulting motifs were compared and for each set of upstream regions (related to a certain TF) an operator region of 16 ('CcpA-like') or 17 ('EbgR-like') bases was defined.

#### - Identification of putative TF binding sites

A specific operator and a position-specific scoring matrix were created for each TF by application of MEME to the defined operator regions. To avoid base preferences in the scoring, a background file in which the probability of finding an A, T, C or G at a certain position at random was set at 0.25. The final position-specific scoring matrices were used as input for an automated genome-wide motif search using MAST. Two additional criteria were used to filter out potential false positives. Firstly, the vast majority of LacI operators that have been identified to date can be found in the range of -250 to +50 nucleotides from the translation start, with no instances further upstream [21,23]. Therefore, identified sites located more than 250 nucleotides upstream and more than 50 nucleotides downstream of the translation start site were not considered. Secondly, all sites that deviated at more than two positions in the central 14 nucleotides with respect to the operators in the vicinity of the LacI-family TFs, were not considered. The tables that resulted from the MAST search have been deposited in Additional file 8.

### Prediction of the inducer of TF activity

A bootstrapped NJ-tree was generated on basis of a multiple sequence alignment of all LacI-family TF homologs of *L. plantarum*, together with orthologous sequences for which experimental confirmation about the nature of the inducer could be retrieved. TFs were considered equivalent in case they were clearly orthologous (strong bootstrap support), were syntenous and provided the alignment was homogeneous (i.e. the absence of gaps and several clear conservations).

### Reconstruction of the mode of regulation

In principle, TFs can act both as transcriptional activator and as repressor depending on the position of the operator relative to the promoter, upstream or inside/downstream, respectively [18,25,58,97]. To resolve whether the TF acts as an activator or repressor, phylogenetic footprints were made for various upstream regions containing an operator and its position relative to that of the potential promoter was determined. In case the alignment was not clear, the predicted operators were used as an anchor to realign the flanking regions for promoter detection.

### Determination of relative mRNA levels for the LacI-family TFs

Absolute expression data was obtained from 35 independent micro-array experiments with custom Agilent oligo-based arrays of *L. plantarum WCFS1 *(this yielded 70 semi-independent datasets). The experimental conditions tested varied from stress to over-expression of certain metabolic genes to growth on different oligosaccharides (D. Molenaar, unpublished data; see also [17]). The raw data were adapted as follows. The absolute signals of the spots related to individual proteins were averaged and then the signals were ranked independently for the two individual channels. Per experiment and per channel, the 50 lowest signals were discarded and the signals of the 200 proteins ranked lowest in the remaining list were averaged. The average was interpreted as basal signal and subtracted from the signals related to the LacI-family TFs. Finally, the resulting signals were made relative by dividing all signals by the highest signal displayed by a LacI-family TF representative.

## Abbreviations

CCR: Carbon Catabolite Repression; CRE: CcpA-Responsive Element; GOOFE: Group of Orthologous Functional Equivalents; TF: Transcription Factor

## Authors' contributions

CF conceived, designed and coordinated the study, carried out the motif and functional analyses and drafted and revised the manuscript; RK conceived and designed the study, carried out the motif analysis, wrote the scripts to convert MEME and MAST output into tabular format and helped revising the manuscript; MW carried out and helped interpret the genome-wide motif searches and helped revising the manuscript; RJS conceived and coordinated the study and helped drafting and revising the manuscript. All authors have read and approved the final manuscript.

## Supplementary Material

Additional file 1 The number of LacI-family members in sequenced Firmicutes. The file lists the species whose genome was studied, their abbreviation and the number of LacI-family TFs recovered for each genome. Click here for file

Additional file 2 IDs and sequences of LacI-family TFs in sequenced Firmicutes. The file contains the sequences that were considered in this study with their different IDs. Click here for file

Additional file 3 Multiple sequence alignment of LacI-family TFs of sequenced Firmicutes. The file gives the sequence alignment of the TFs considered in this study. Click here for file

Additional file 4 Neighbor Joining tree for the LacI-family TFs of sequenced Firmicutes. The file gives the bootstrapped (n = 250) NJ-tree for the LacI-family TF homologs of the *Firmicutes*, *Salmonella *and *E. coli*. Click here for file

Additional file 5 Multiple sequence alignments and Neighbor joining trees for the two functional domains of the LacI-family TF homologs in *L. plantarum*. The file contains images of the sequence alignments and the bootstrapped (n = 1000) NJ-trees for the two TF functional domains. Click here for file

Additional file 6 Gene context conservation of the LacI-family TF homologs in *L. plantarum *WCFS1. The file provides a visualization of context information that was used to define the GOOFEs and contains the motifs used to perform the MAST searches. Click here for file

Additional file 7 LacI-family homologs deviant at position 24 and their putative operators. The file lists the putative binding motifs for several LacITFs with a conserved substitution of the conserved arginine at position 24 (in Figure 2). Click here for file

Additional file 8 MAST output. The file contains the parsed output of the MAST searches. A new sheet is provided for each criterion that was applied to constrain the list. The results are color-coded. Click here for file

Additional file 9 The functional annotation of the genes and operons regulated by LacI-TFs in *L. plantarum*. The file contains a functional description of the genes and operons that putatively constitute the minimal regulons depicted in Figure 3 (with relevant references). Click here for file
