# Supplementary material for: A generic approach to identify Transcription Factor-specific operator motifs; Inferences for LacI-family mediated regulation in Lactobacillus plantarum WCFS1
Source: BMC Genomics. 2008 Mar 27;9:145. doi: 10.1186/1471-2164-9-145 (PMC2329647; doi:10.1186/1471-2164-9-145)
Supplement: Additional file 5 — Multiple sequence alignments and Neighbor joining trees for the two functional domains of the LacI-family TF homologs in L. plantarum. The file contains images of the sequence alignments and the bootstrapped (n = 1000) NJ-trees for the two TF functional domains. [file 1471-2164-9-145-S5.ppt]

## Slide 1
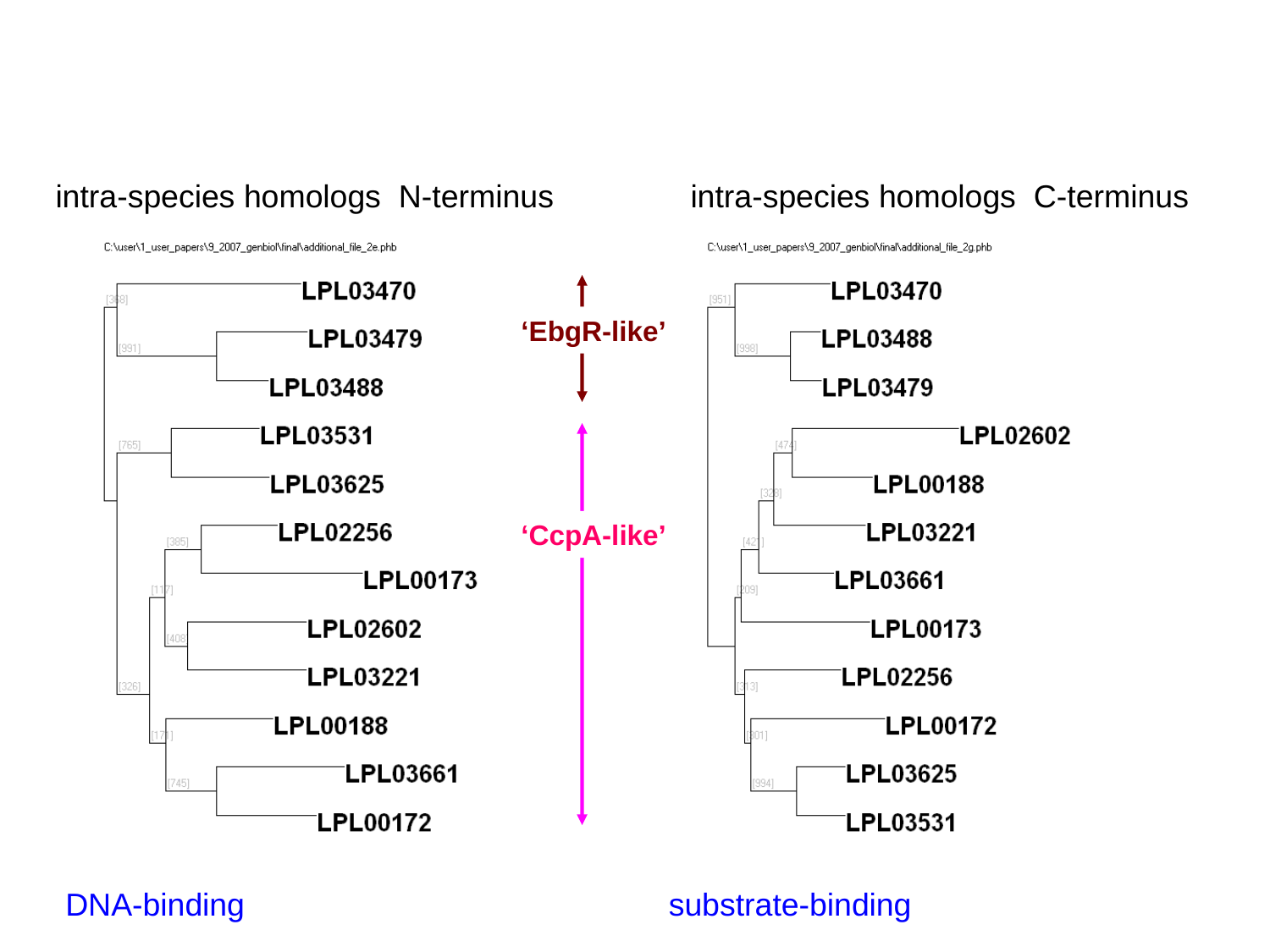

intra-species homologs N-terminus
intra-species homologs C-terminus
‘EbgR-like’
‘CcpA-like’
DNA-binding
substrate-binding

## Slide 2
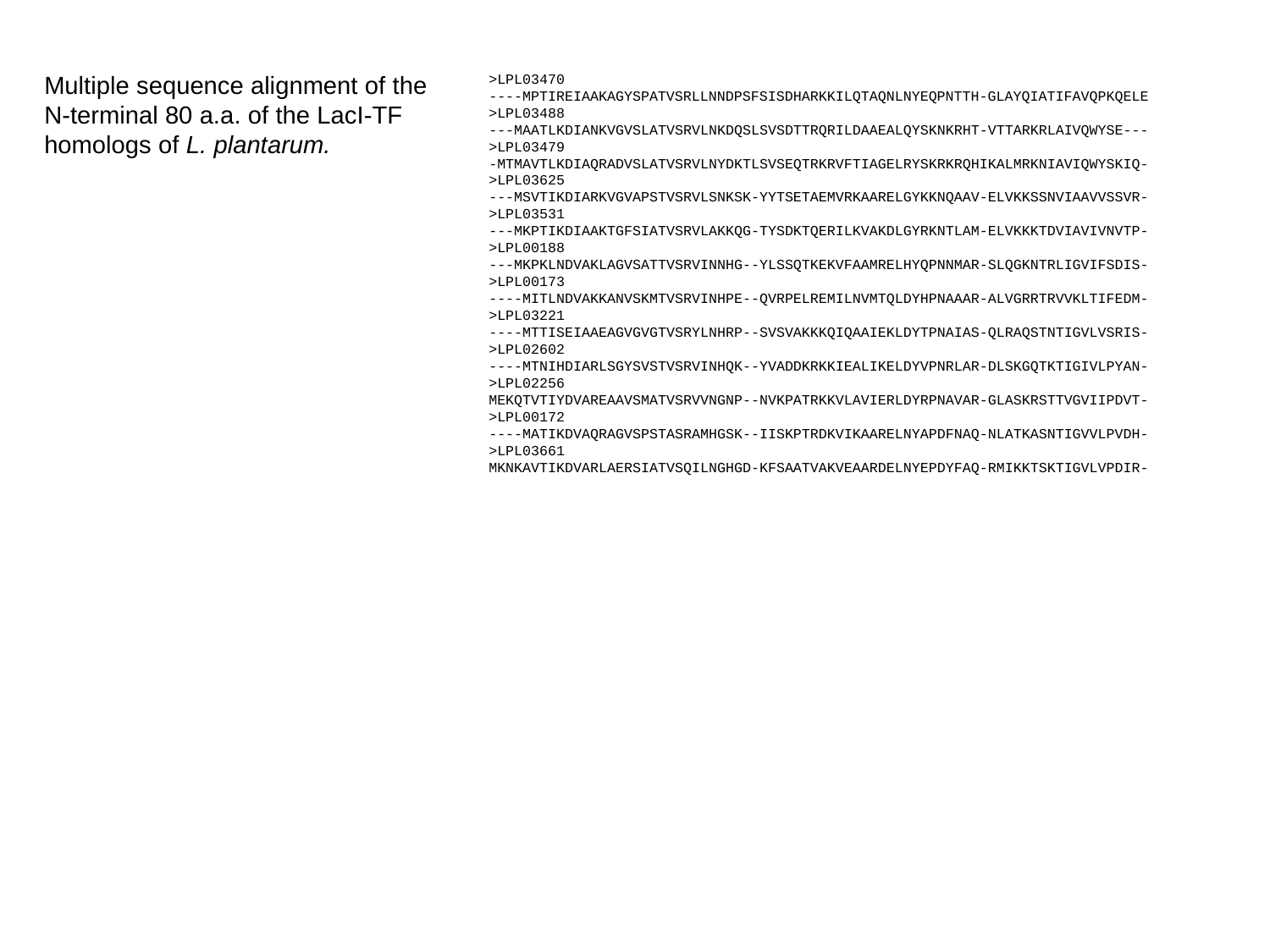

Multiple sequence alignment of the N-terminal 80 a.a. of the LacI-TF homologs of L. plantarum.
>LPL03470
----MPTIREIAAKAGYSPATVSRLLNNDPSFSISDHARKKILQTAQNLNYEQPNTTH-GLAYQIATIFAVQPKQELE
>LPL03488
---MAATLKDIANKVGVSLATVSRVLNKDQSLSVSDTTRQRILDAAEALQYSKNKRHT-VTTARKRLAIVQWYSE---
>LPL03479
-MTMAVTLKDIAQRADVSLATVSRVLNYDKTLSVSEQTRKRVFTIAGELRYSKRKRQHIKALMRKNIAVIQWYSKIQ-
>LPL03625
---MSVTIKDIARKVGVAPSTVSRVLSNKSK-YYTSETAEMVRKAARELGYKKNQAAV-ELVKKSSNVIAAVVSSVR-
>LPL03531
---MKPTIKDIAAKTGFSIATVSRVLAKKQG-TYSDKTQERILKVAKDLGYRKNTLAM-ELVKKKTDVIAVIVNVTP-
>LPL00188
---MKPKLNDVAKLAGVSATTVSRVINNHG--YLSSQTKEKVFAAMRELHYQPNNMAR-SLQGKNTRLIGVIFSDIS-
>LPL00173
----MITLNDVAKKANVSKMTVSRVINHPE--QVRPELREMILNVMTQLDYHPNAAAR-ALVGRRTRVVKLTIFEDM-
>LPL03221
----MTTISEIAAEAGVGVGTVSRYLNHRP--SVSVAKKKQIQAAIEKLDYTPNAIAS-QLRAQSTNTIGVLVSRIS-
>LPL02602
----MTNIHDIARLSGYSVSTVSRVINHQK--YVADDKRKKIEALIKELDYVPNRLAR-DLSKGQTKTIGIVLPYAN-
>LPL02256
MEKQTVTIYDVAREAAVSMATVSRVVNGNP--NVKPATRKKVLAVIERLDYRPNAVAR-GLASKRSTTVGVIIPDVT-
>LPL00172
----MATIKDVAQRAGVSPSTASRAMHGSK--IISKPTRDKVIKAARELNYAPDFNAQ-NLATKASNTIGVVLPVDH-
>LPL03661
MKNKAVTIKDVARLAERSIATVSQILNGHGD-KFSAATVAKVEAARDELNYEPDYFAQ-RMIKKTSKTIGVLVPDIR-

## Slide 3
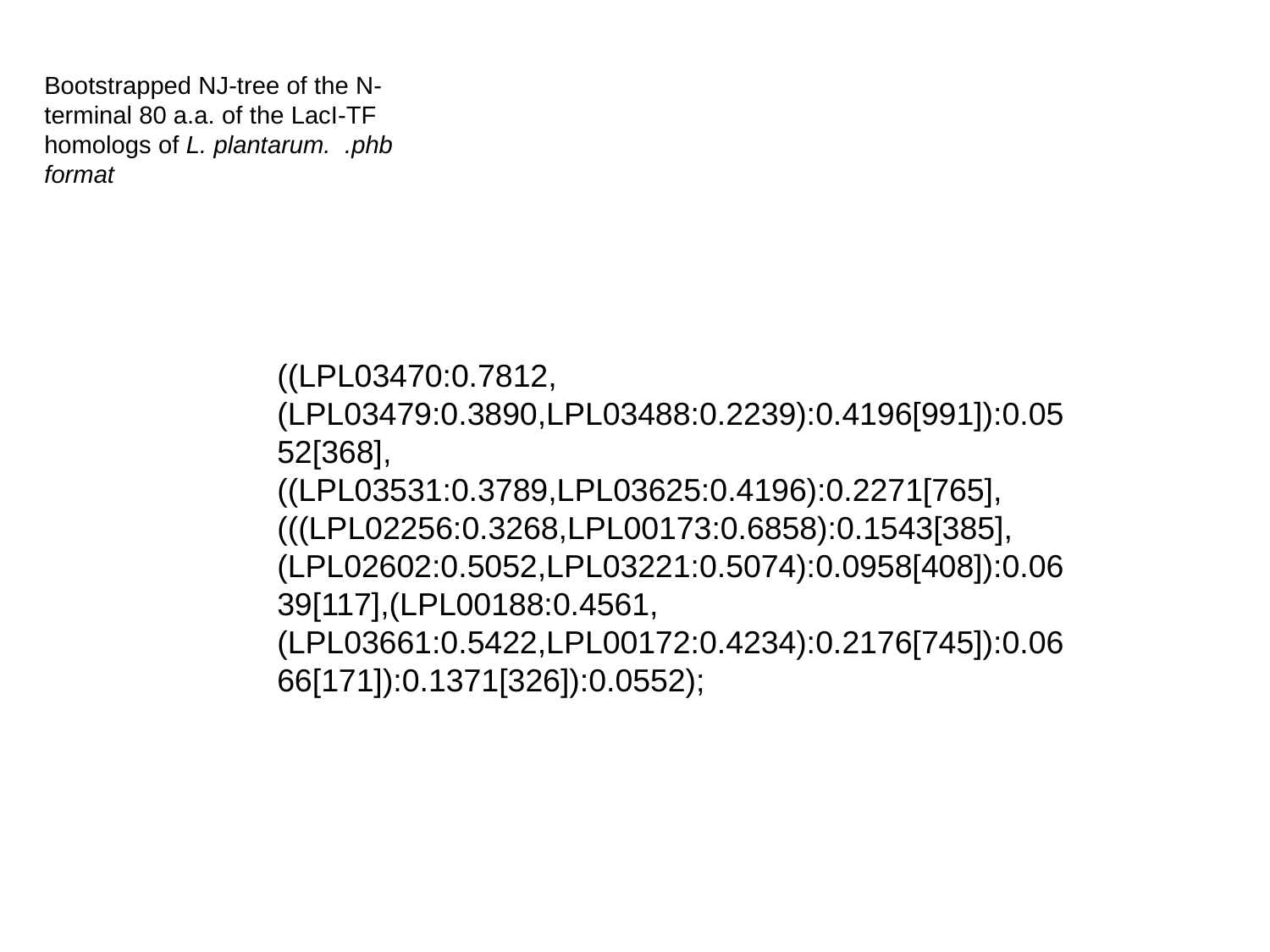

Bootstrapped NJ-tree of the N-terminal 80 a.a. of the LacI-TF homologs of L. plantarum. .phb format
((LPL03470:0.7812,(LPL03479:0.3890,LPL03488:0.2239):0.4196[991]):0.0552[368],((LPL03531:0.3789,LPL03625:0.4196):0.2271[765],(((LPL02256:0.3268,LPL00173:0.6858):0.1543[385],(LPL02602:0.5052,LPL03221:0.5074):0.0958[408]):0.0639[117],(LPL00188:0.4561,(LPL03661:0.5422,LPL00172:0.4234):0.2176[745]):0.0666[171]):0.1371[326]):0.0552);

## Slide 4
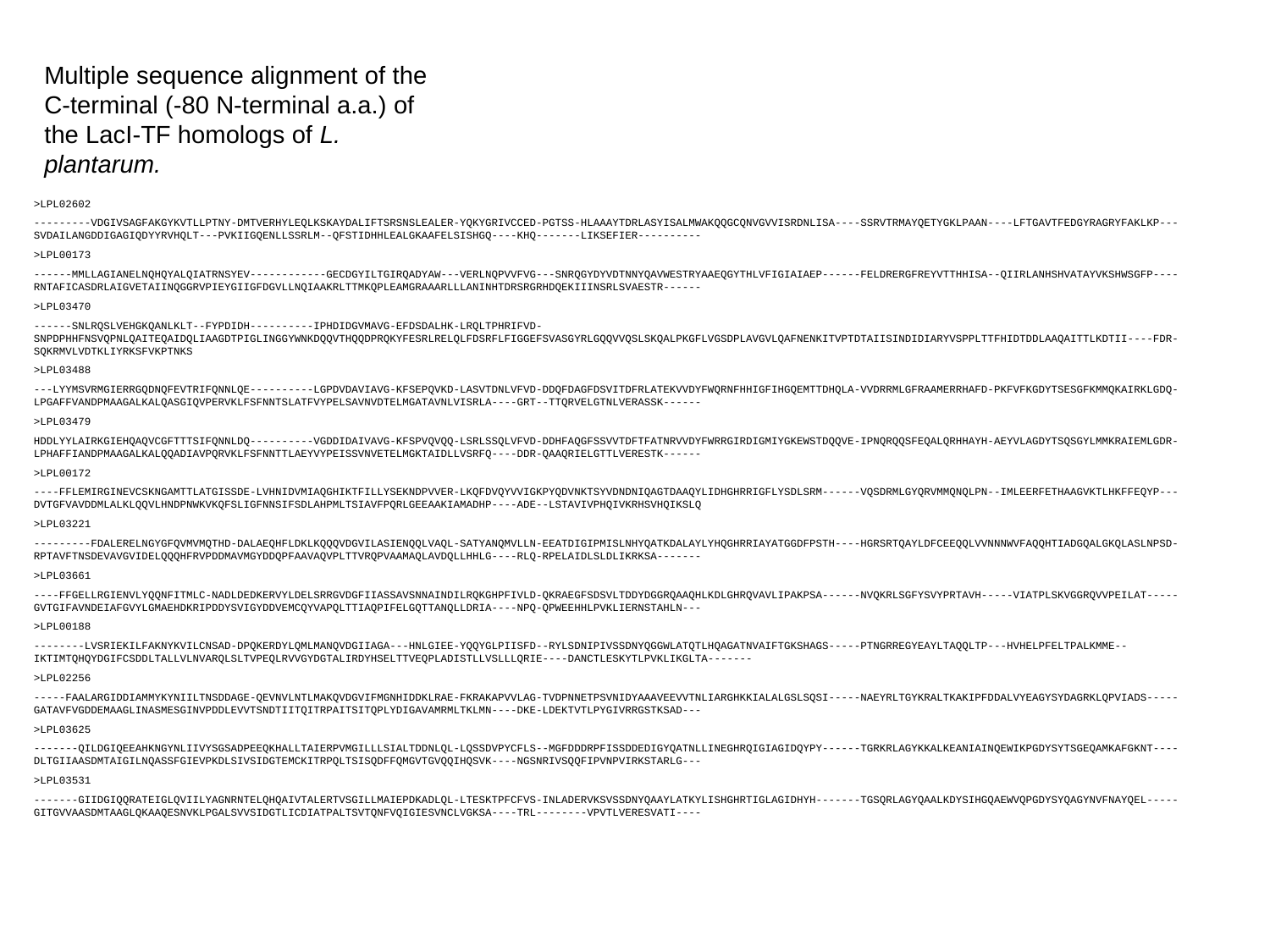

Multiple sequence alignment of the C-terminal (-80 N-terminal a.a.) of the LacI-TF homologs of L. plantarum.
>LPL02602
---------VDGIVSAGFAKGYKVTLLPTNY-DMTVERHYLEQLKSKAYDALIFTSRSNSLEALER-YQKYGRIVCCED-PGTSS-HLAAAYTDRLASYISALMWAKQQGCQNVGVVISRDNLISA----SSRVTRMAYQETYGKLPAAN----LFTGAVTFEDGYRAGRYFAKLKP---SVDAILANGDDIGAGIQDYYRVHQLT---PVKIIGQENLLSSRLM--QFSTIDHHLEALGKAAFELSISHGQ----KHQ-------LIKSEFIER----------
>LPL00173
------MMLLAGIANELNQHQYALQIATRNSYEV------------GECDGYILTGIRQADYAW---VERLNQPVVFVG---SNRQGYDYVDTNNYQAVWESTRYAAEQGYTHLVFIGIAIAEP------FELDRERGFREYVTTHHISA--QIIRLANHSHVATAYVKSHWSGFP----RNTAFICASDRLAIGVETAIINQGGRVPIEYGIIGFDGVLLNQIAAKRLTTMKQPLEAMGRAAARLLLANINHTDRSRGRHDQEKIIINSRLSVAESTR------
>LPL03470
------SNLRQSLVEHGKQANLKLT--FYPDIDH----------IPHDIDGVMAVG-EFDSDALHK-LRQLTPHRIFVD-SNPDPHHFNSVQPNLQAITEQAIDQLIAAGDTPIGLINGGYWNKDQQVTHQQDPRQKYFESRLRELQLFDSRFLFIGGEFSVASGYRLGQQVVQSLSKQALPKGFLVGSDPLAVGVLQAFNENKITVPTDTAIISINDIDIARYVSPPLTTFHIDTDDLAAQAITTLKDTII----FDR-SQKRMVLVDTKLIYRKSFVKPTNKS
>LPL03488
---LYYMSVRMGIERRGQDNQFEVTRIFQNNLQE----------LGPDVDAVIAVG-KFSEPQVKD-LASVTDNLVFVD-DDQFDAGFDSVITDFRLATEKVVDYFWQRNFHHIGFIHGQEMTTDHQLA-VVDRRMLGFRAAMERRHAFD-PKFVFKGDYTSESGFKMMQKAIRKLGDQ-LPGAFFVANDPMAAGALKALQASGIQVPERVKLFSFNNTSLATFVYPELSAVNVDTELMGATAVNLVISRLA----GRT--TTQRVELGTNLVERASSK------
>LPL03479
HDDLYYLAIRKGIEHQAQVCGFTTTSIFQNNLDQ----------VGDDIDAIVAVG-KFSPVQVQQ-LSRLSSQLVFVD-DDHFAQGFSSVVTDFTFATNRVVDYFWRRGIRDIGMIYGKEWSTDQQVE-IPNQRQQSFEQALQRHHAYH-AEYVLAGDYTSQSGYLMMKRAIEMLGDR-LPHAFFIANDPMAAGALKALQQADIAVPQRVKLFSFNNTTLAEYVYPEISSVNVETELMGKTAIDLLVSRFQ----DDR-QAAQRIELGTTLVERESTK------
>LPL00172
----FFLEMIRGINEVCSKNGAMTTLATGISSDE-LVHNIDVMIAQGHIKTFILLYSEKNDPVVER-LKQFDVQYVVIGKPYQDVNKTSYVDNDNIQAGTDAAQYLIDHGHRRIGFLYSDLSRM------VQSDRMLGYQRVMMQNQLPN--IMLEERFETHAAGVKTLHKFFEQYP---DVTGFVAVDDMLALKLQQVLHNDPNWKVKQFSLIGFNNSIFSDLAHPMLTSIAVFPQRLGEEAAKIAMADHP----ADE--LSTAVIVPHQIVKRHSVHQIKSLQ
>LPL03221
---------FDALERELNGYGFQVMVMQTHD-DALAEQHFLDKLKQQQVDGVILASIENQQLVAQL-SATYANQMVLLN-EEATDIGIPMISLNHYQATKDALAYLYHQGHRRIAYATGGDFPSTH----HGRSRTQAYLDFCEEQQLVVNNNWVFAQQHTIADGQALGKQLASLNPSD-RPTAVFTNSDEVAVGVIDELQQQHFRVPDDMAVMGYDDQPFAAVAQVPLTTVRQPVAAMAQLAVDQLLHHLG----RLQ-RPELAIDLSLDLIKRKSA-------
>LPL03661
----FFGELLRGIENVLYQQNFITMLC-NADLDEDKERVYLDELSRRGVDGFIIASSAVSNNAINDILRQKGHPFIVLD-QKRAEGFSDSVLTDDYDGGRQAAQHLKDLGHRQVAVLIPAKPSA------NVQKRLSGFYSVYPRTAVH-----VIATPLSKVGGRQVVPEILAT-----GVTGIFAVNDEIAFGVYLGMAEHDKRIPDDYSVIGYDDVEMCQYVAPQLTTIAQPIFELGQTTANQLLDRIA----NPQ-QPWEEHHLPVKLIERNSTAHLN---
>LPL00188
--------LVSRIEKILFAKNYKVILCNSAD-DPQKERDYLQMLMANQVDGIIAGA---HNLGIEE-YQQYGLPIISFD--RYLSDNIPIVSSDNYQGGWLATQTLHQAGATNVAIFTGKSHAGS-----PTNGRREGYEAYLTAQQLTP---HVHELPFELTPALKMME--IKTIMTQHQYDGIFCSDDLTALLVLNVARQLSLTVPEQLRVVGYDGTALIRDYHSELTTVEQPLADISTLLVSLLLQRIE----DANCTLESKYTLPVKLIKGLTA-------
>LPL02256
-----FAALARGIDDIAMMYKYNIILTNSDDAGE-QEVNVLNTLMAKQVDGVIFMGNHIDDKLRAE-FKRAKAPVVLAG-TVDPNNETPSVNIDYAAAVEEVVTNLIARGHKKIALALGSLSQSI-----NAEYRLTGYKRALTKAKIPFDDALVYEAGYSYDAGRKLQPVIADS-----GATAVFVGDDEMAAGLINASMESGINVPDDLEVVTSNDTIITQITRPAITSITQPLYDIGAVAMRMLTKLMN----DKE-LDEKTVTLPYGIVRRGSTKSAD---
>LPL03625
-------QILDGIQEEAHKNGYNLIIVYSGSADPEEQKHALLTAIERPVMGILLLSIALTDDNLQL-LQSSDVPYCFLS--MGFDDDRPFISSDDEDIGYQATNLLINEGHRQIGIAGIDQYPY------TGRKRLAGYKKALKEANIAINQEWIKPGDYSYTSGEQAMKAFGKNT----DLTGIIAASDMTAIGILNQASSFGIEVPKDLSIVSIDGTEMCKITRPQLTSISQDFFQMGVTGVQQIHQSVK----NGSNRIVSQQFIPVNPVIRKSTARLG---
>LPL03531
-------GIIDGIQQRATEIGLQVIILYAGNRNTELQHQAIVTALERTVSGILLMAIEPDKADLQL-LTESKTPFCFVS-INLADERVKSVSSDNYQAAYLATKYLISHGHRTIGLAGIDHYH-------TGSQRLAGYQAALKDYSIHGQAEWVQPGDYSYQAGYNVFNAYQEL-----GITGVVAASDMTAAGLQKAAQESNVKLPGALSVVSIDGTLICDIATPALTSVTQNFVQIGIESVNCLVGKSA----TRL--------VPVTLVERESVATI----

## Slide 5
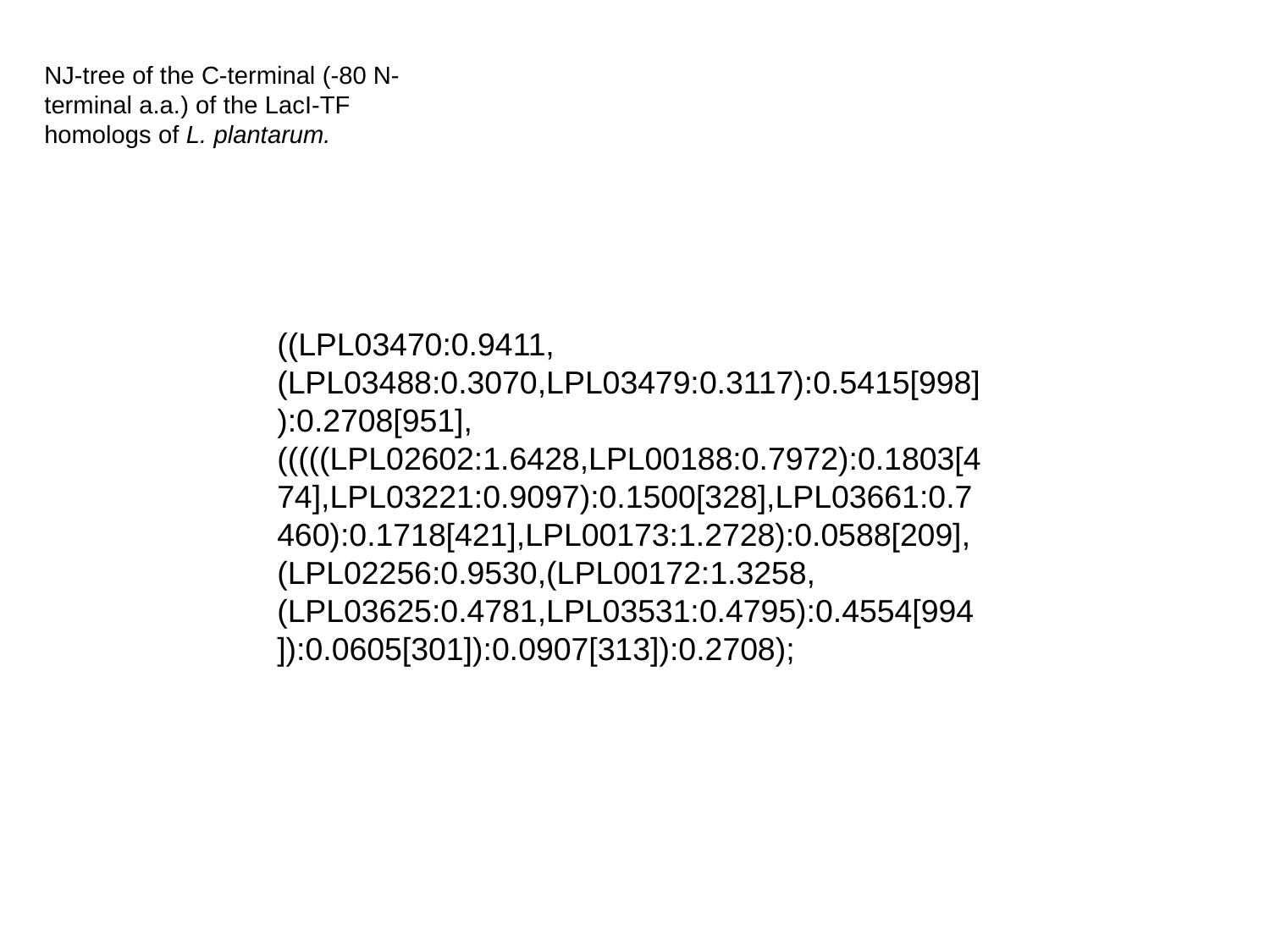

NJ-tree of the C-terminal (-80 N-terminal a.a.) of the LacI-TF homologs of L. plantarum.
((LPL03470:0.9411,(LPL03488:0.3070,LPL03479:0.3117):0.5415[998]):0.2708[951],(((((LPL02602:1.6428,LPL00188:0.7972):0.1803[474],LPL03221:0.9097):0.1500[328],LPL03661:0.7460):0.1718[421],LPL00173:1.2728):0.0588[209],(LPL02256:0.9530,(LPL00172:1.3258,(LPL03625:0.4781,LPL03531:0.4795):0.4554[994]):0.0605[301]):0.0907[313]):0.2708);
