# Supplementary material for: A generic approach to identify Transcription Factor-specific operator motifs; Inferences for LacI-family mediated regulation in Lactobacillus plantarum WCFS1
Source: BMC Genomics. 2008 Mar 27;9:145. doi: 10.1186/1471-2164-9-145 (PMC2329647; doi:10.1186/1471-2164-9-145)
Supplement: Additional file 6 — Gene context conservation of the LacI-family TF homologs in L. plantarum WCFS1. The file provides a visualization of context information that was used to define the GOOFEs and contains the motifs used to perform the MAST searches. [file 1471-2164-9-145-S6.ppt]

## Slide 1
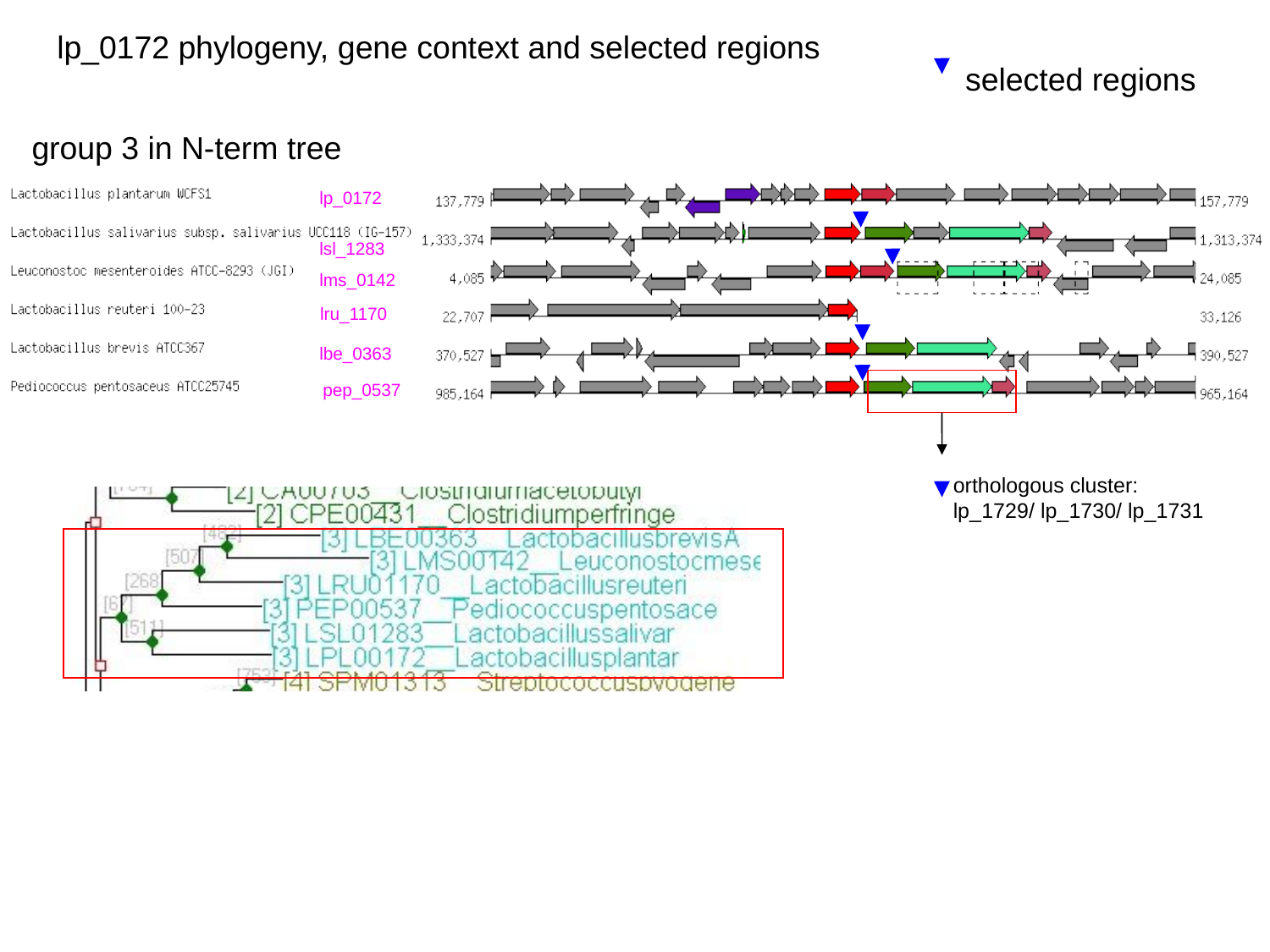

lp_0172 phylogeny, gene context and selected regions
selected regions
group 3 in N-term tree
lp_0172
lsl_1283
lms_0142
lru_1170
lbe_0363
pep_0537
orthologous cluster:
lp_1729/ lp_1730/ lp_1731

## Slide 2
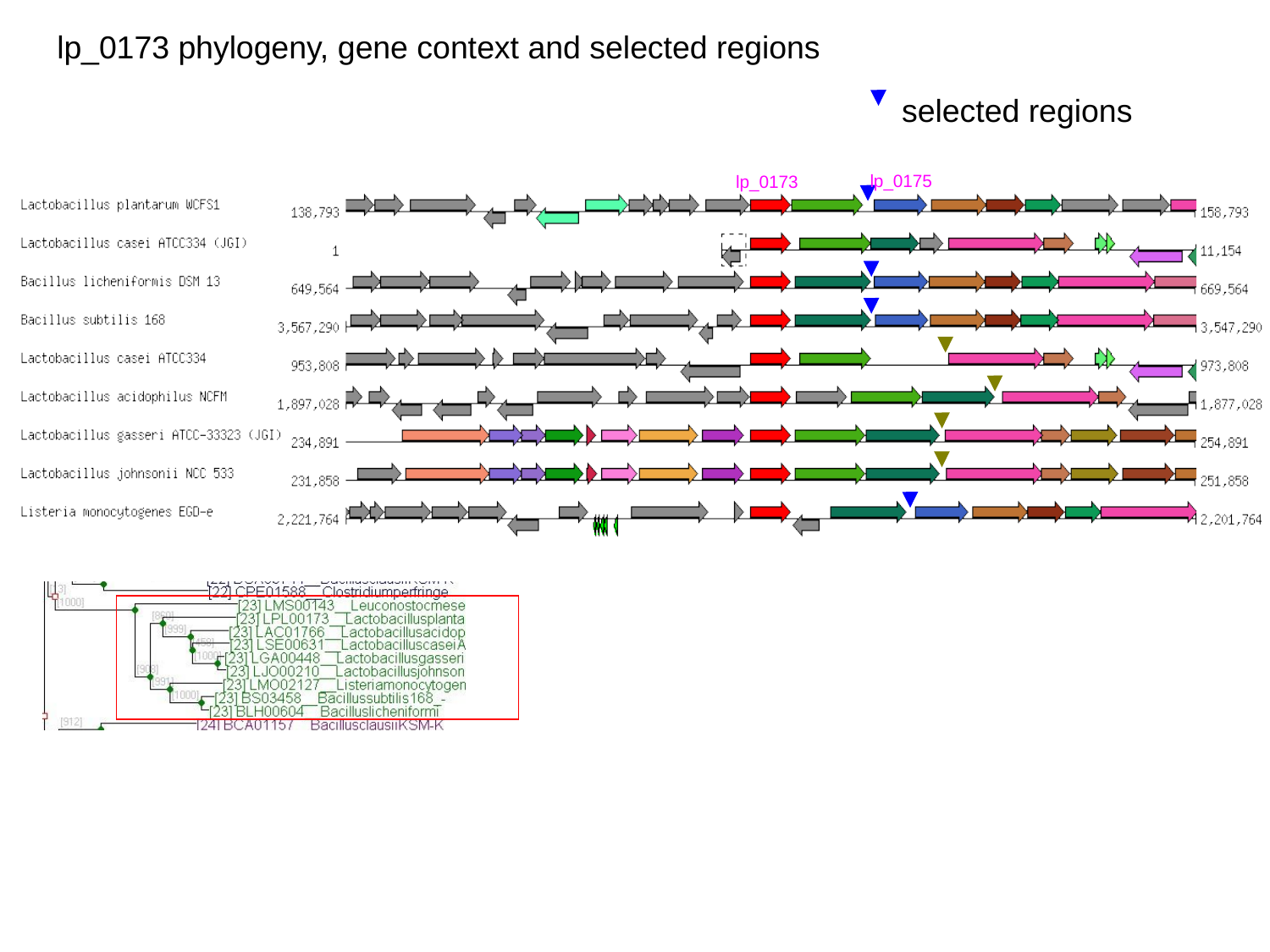

lp_0173 phylogeny, gene context and selected regions
selected regions
lp_0175
lp_0173

## Slide 3
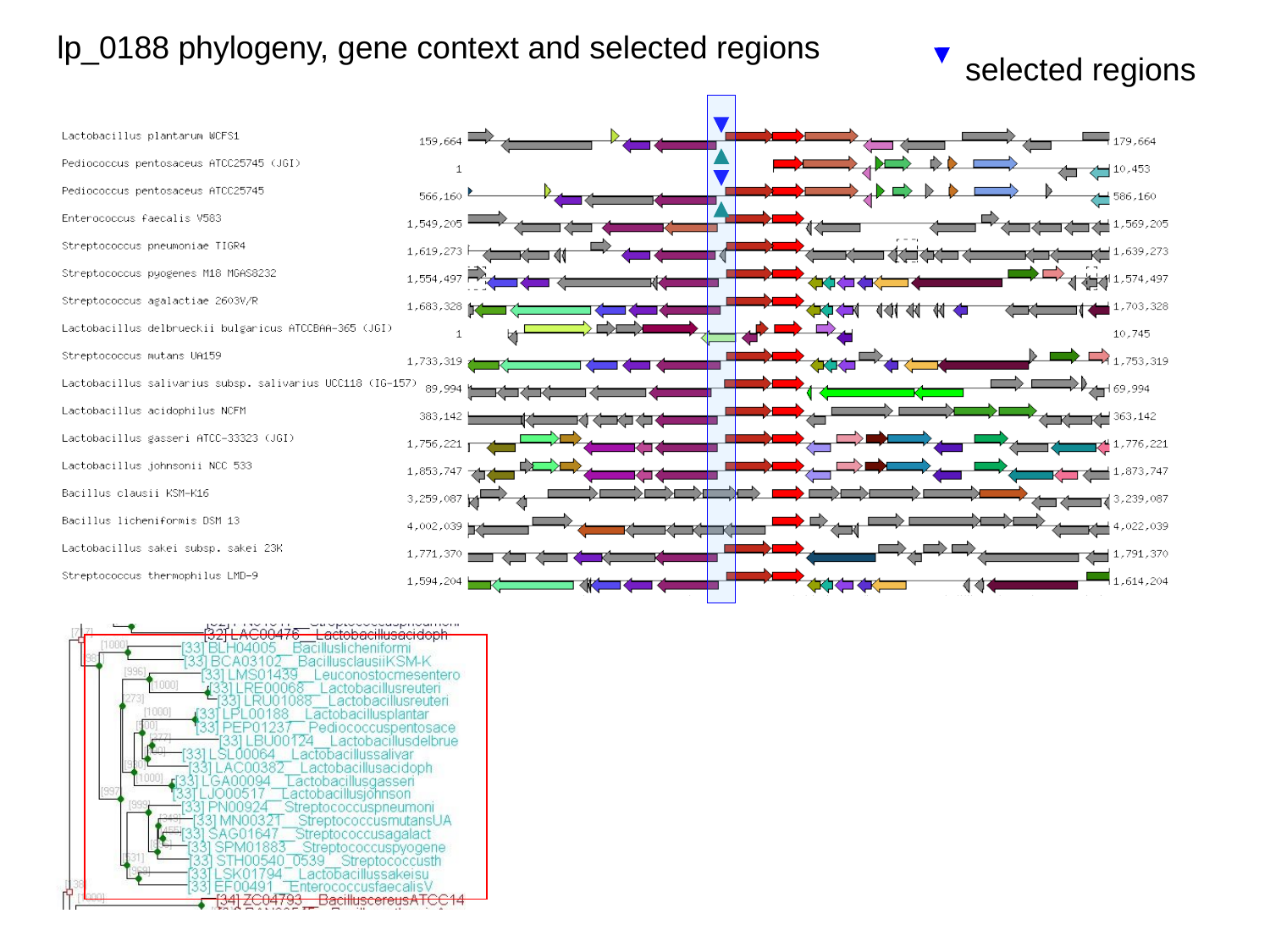

lp_0188 phylogeny, gene context and selected regions
selected regions

## Slide 4
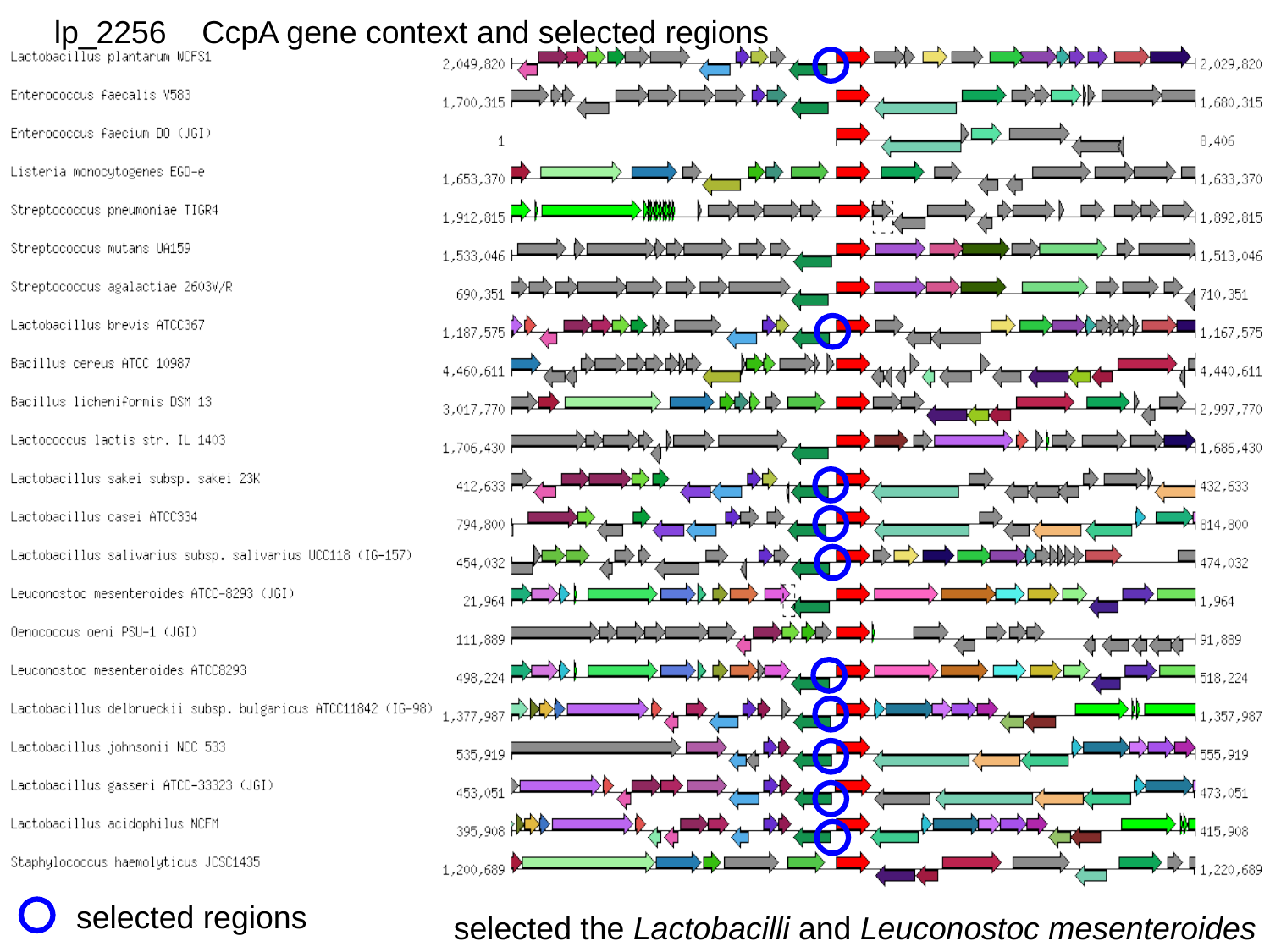

lp_2256 CcpA gene context and selected regions
selected regions
selected the Lactobacilli and Leuconostoc mesenteroides

## Slide 5
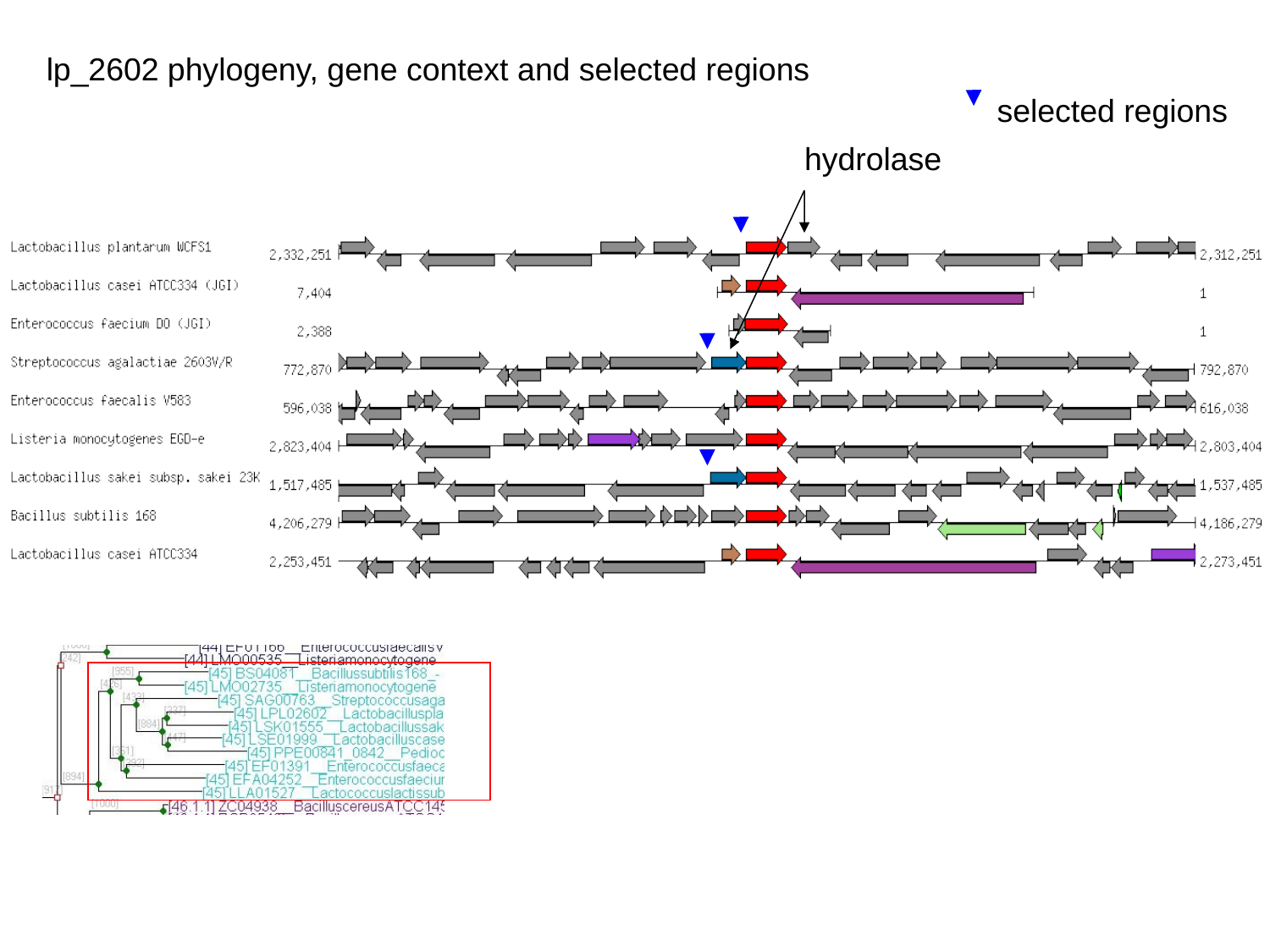

lp_2602 phylogeny, gene context and selected regions
selected regions
hydrolase

## Slide 6
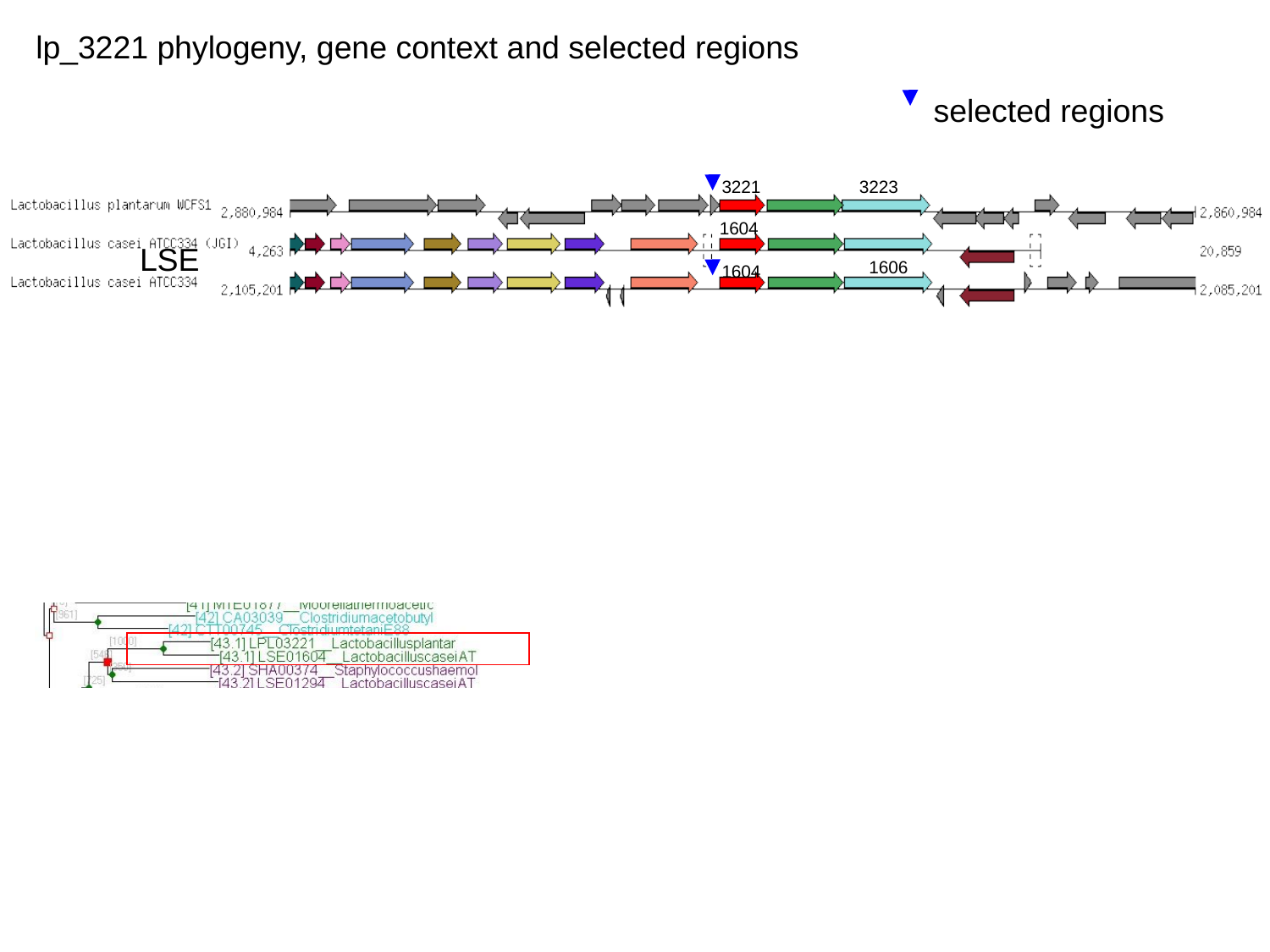

lp_3221 phylogeny, gene context and selected regions
selected regions
3221
3223
1604
LSE
1606
1604

## Slide 7
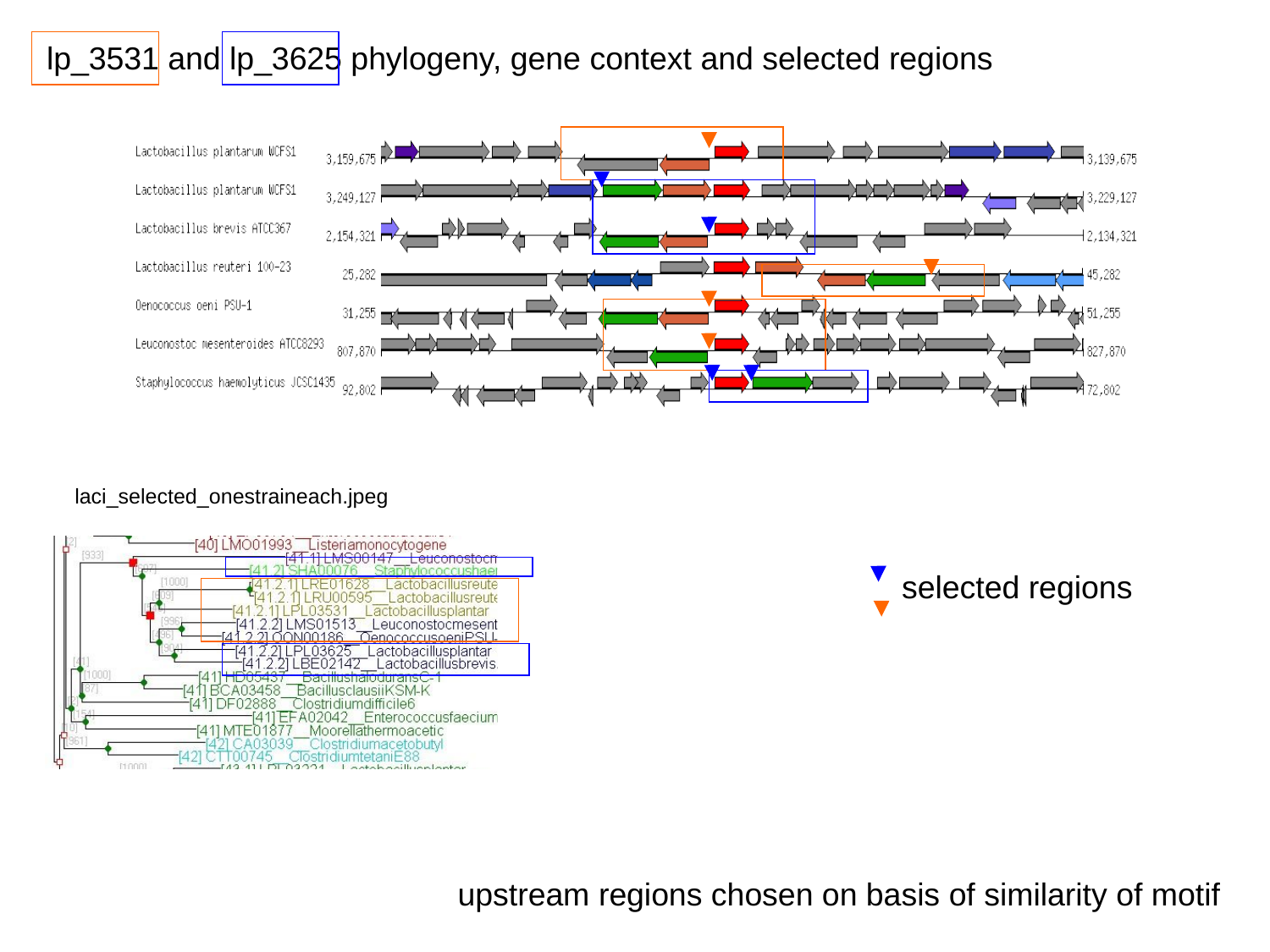

lp_3531 and lp_3625 phylogeny, gene context and selected regions
laci_selected_onestraineach.jpeg
selected regions
upstream regions chosen on basis of similarity of motif

## Slide 8
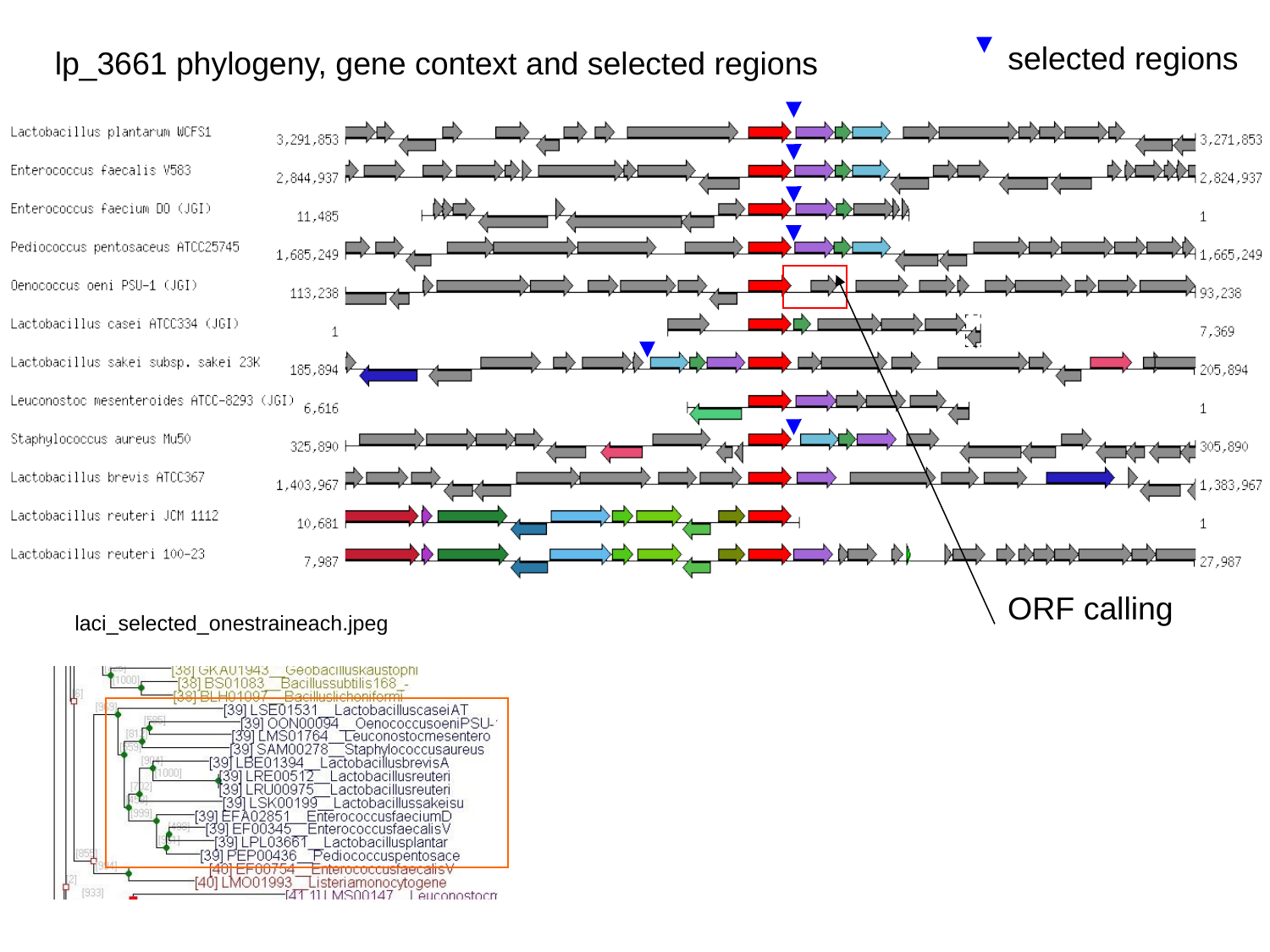

selected regions
lp_3661 phylogeny, gene context and selected regions
ORF calling
laci_selected_onestraineach.jpeg

## Slide 9
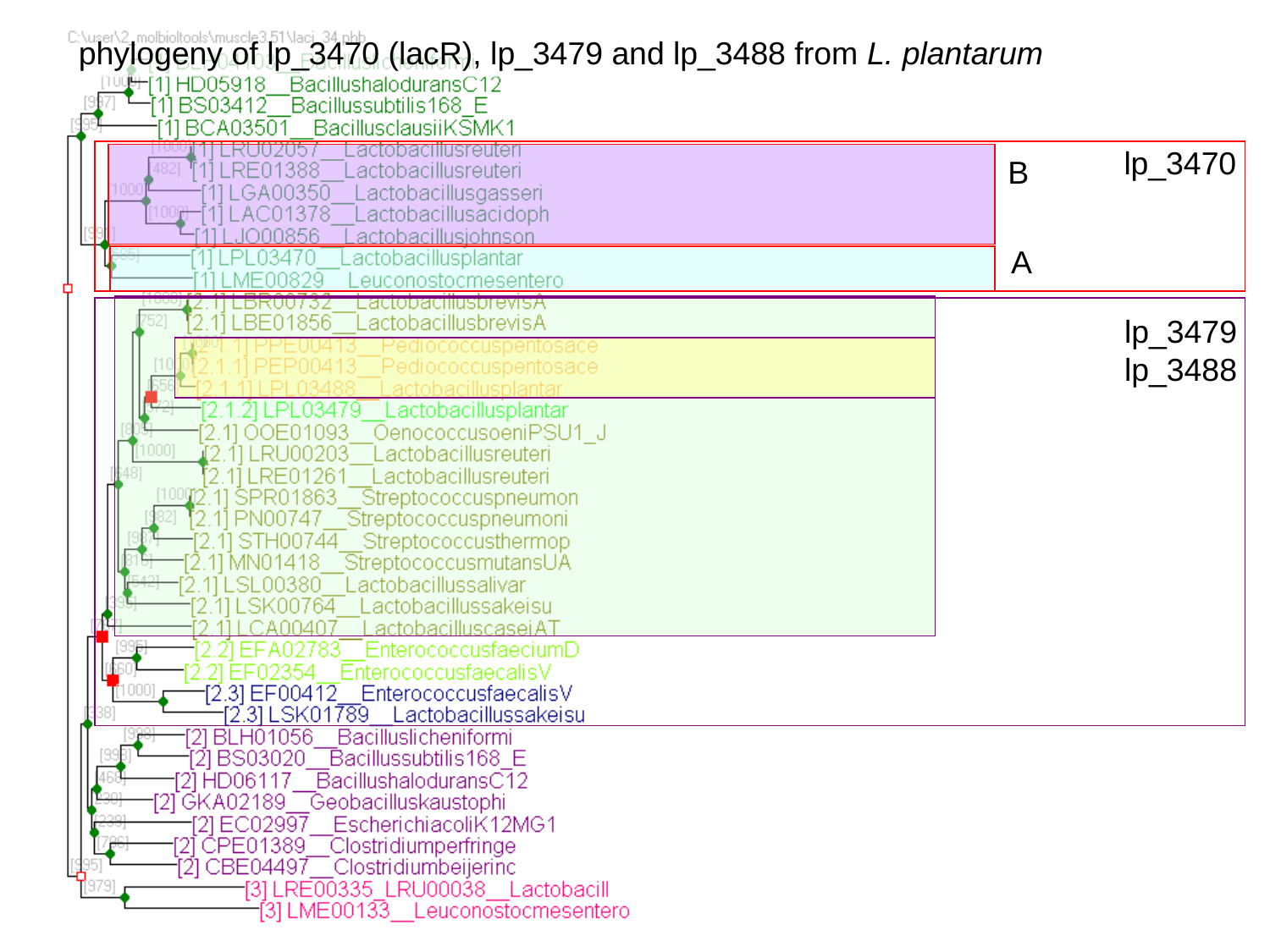

phylogeny of lp_3470 (lacR), lp_3479 and lp_3488 from L. plantarum
lp_3470
B
A
lp_3479
lp_3488

## Slide 10
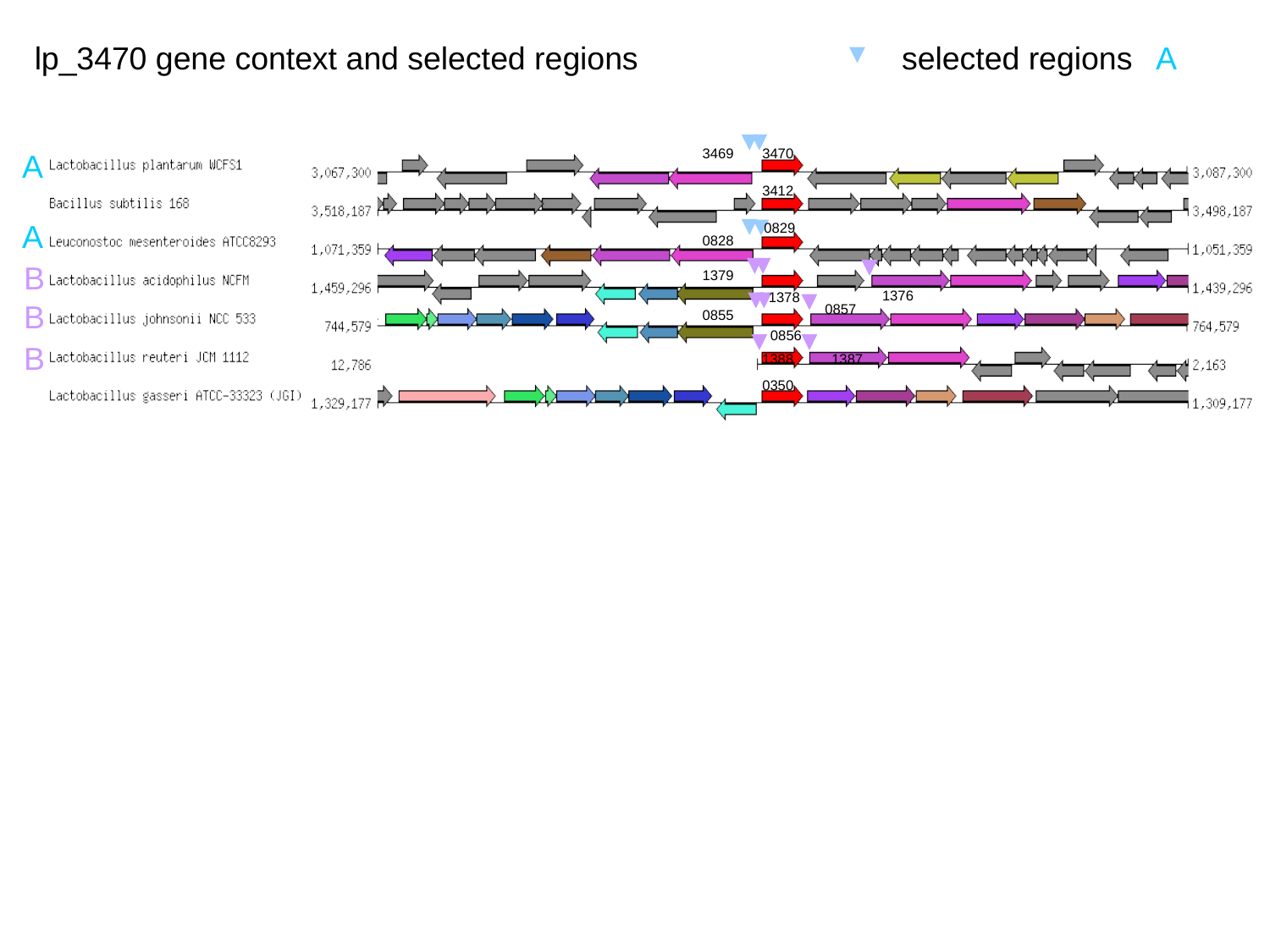

lp_3470 gene context and selected regions
selected regions
A
3469
3470
A
3412
A
0829
0828
B
1379
1376
1378
B
0857
0855
0856
B
1388
1387
0350

## Slide 11
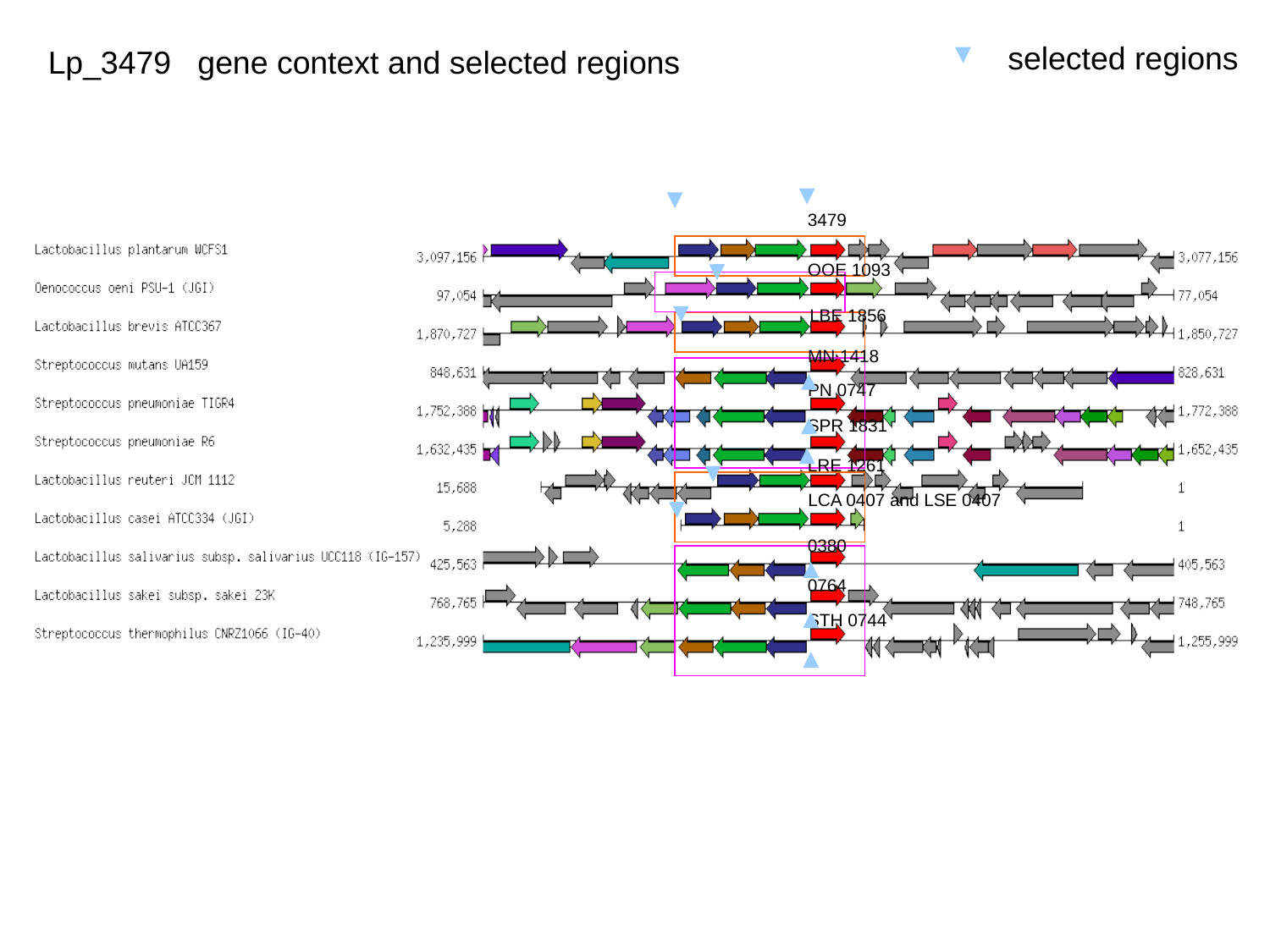

selected regions
Lp_3479 gene context and selected regions
3479
OOE 1093
LBE 1856
MN 1418
PN 0747
SPR 1831
LRE 1261
LCA 0407 and LSE 0407
0380
0764
STH 0744

## Slide 12
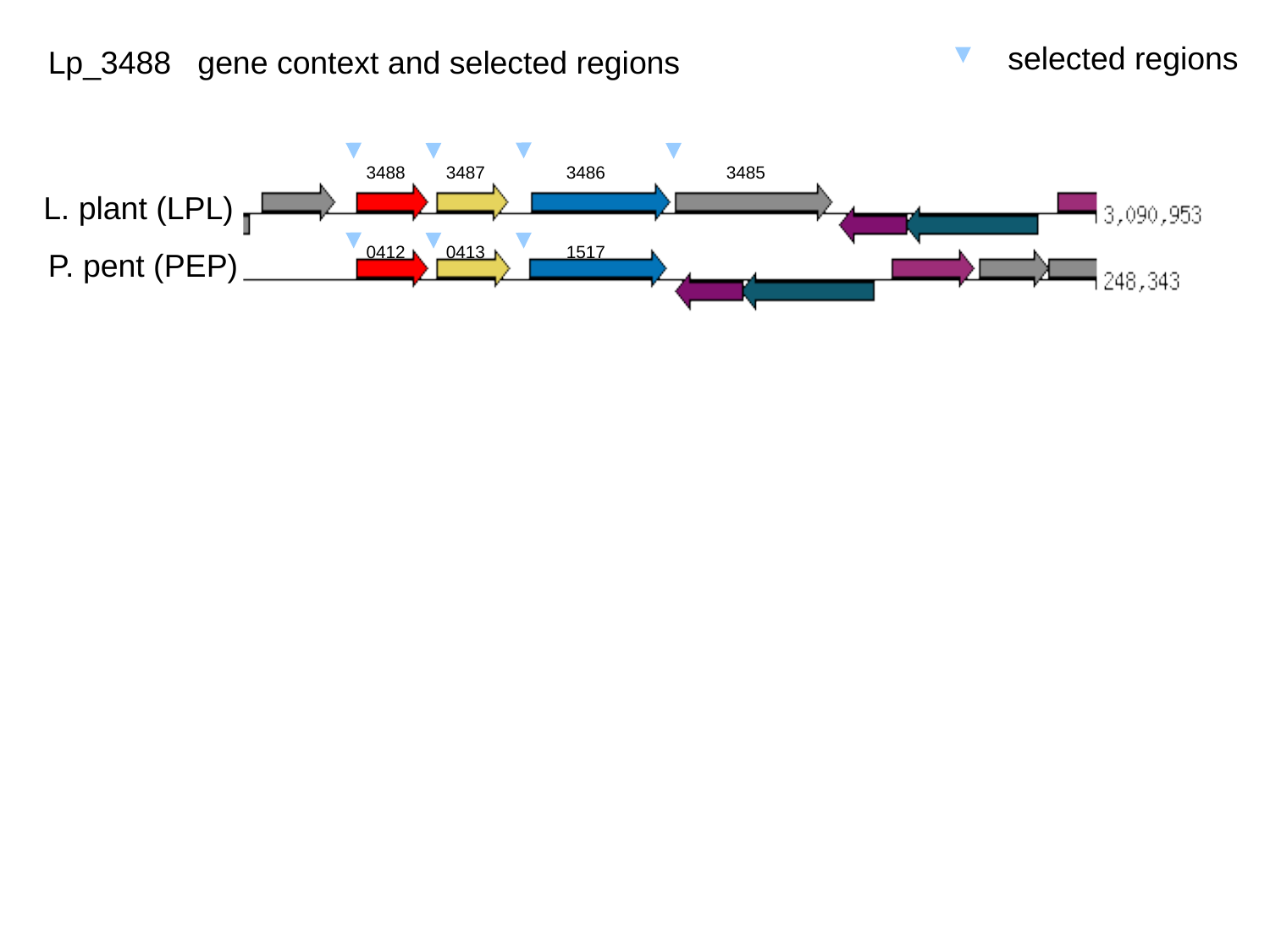

selected regions
Lp_3488 gene context and selected regions
3485
3488
3487
3486
L. plant (LPL)
0412
0413
1517
P. pent (PEP)

## Slide 13
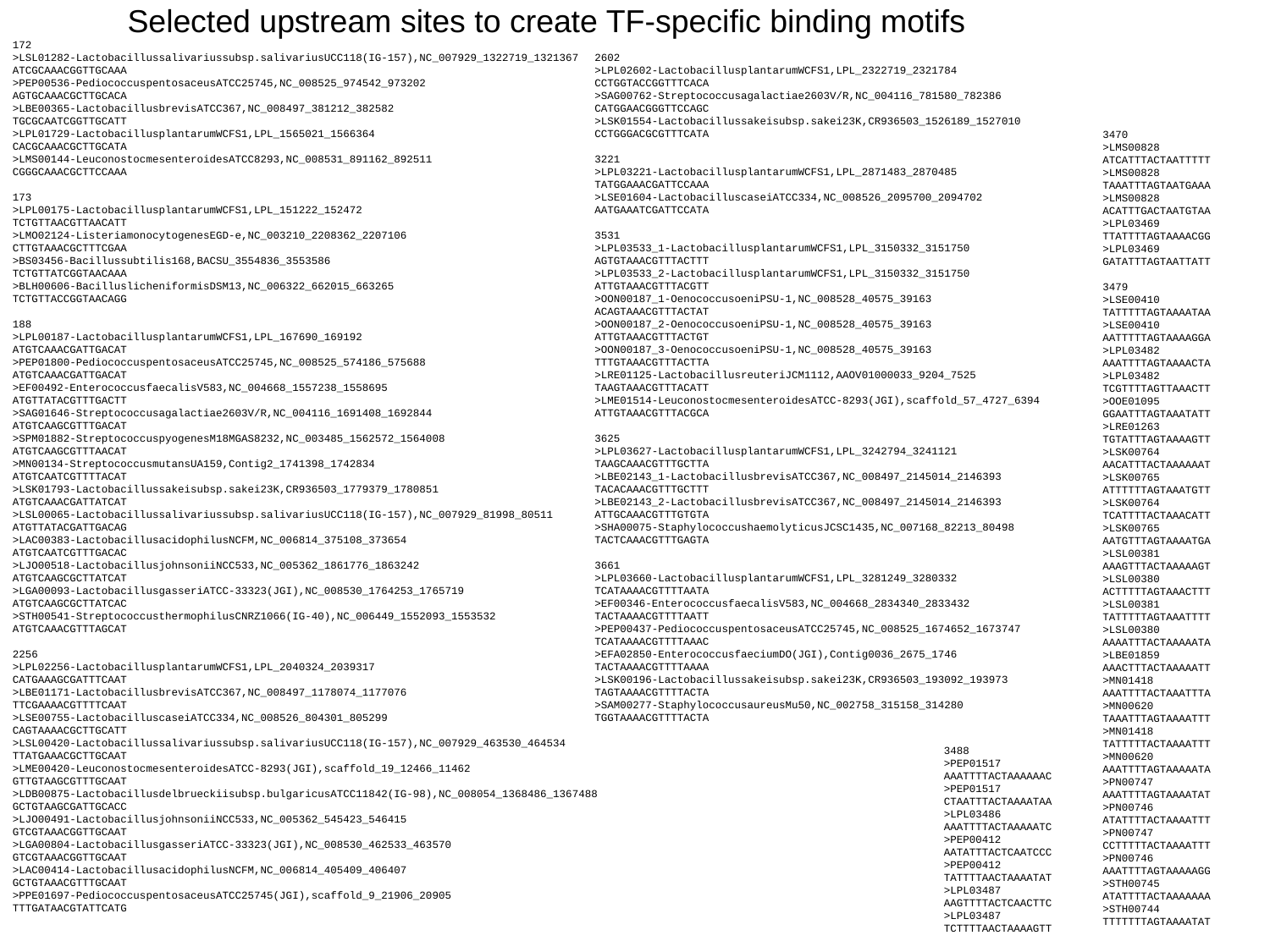

Selected upstream sites to create TF-specific binding motifs
172
>LSL01282-Lactobacillussalivariussubsp.salivariusUCC118(IG-157),NC_007929_1322719_1321367
ATCGCAAACGGTTGCAAA
>PEP00536-PediococcuspentosaceusATCC25745,NC_008525_974542_973202
AGTGCAAACGCTTGCACA
>LBE00365-LactobacillusbrevisATCC367,NC_008497_381212_382582
TGCGCAATCGGTTGCATT
>LPL01729-LactobacillusplantarumWCFS1,LPL_1565021_1566364
CACGCAAACGCTTGCATA
>LMS00144-LeuconostocmesenteroidesATCC8293,NC_008531_891162_892511
CGGGCAAACGCTTCCAAA
173
>LPL00175-LactobacillusplantarumWCFS1,LPL_151222_152472
TCTGTTAACGTTAACATT
>LMO02124-ListeriamonocytogenesEGD-e,NC_003210_2208362_2207106
CTTGTAAACGCTTTCGAA
>BS03456-Bacillussubtilis168,BACSU_3554836_3553586
TCTGTTATCGGTAACAAA
>BLH00606-BacilluslicheniformisDSM13,NC_006322_662015_663265
TCTGTTACCGGTAACAGG
188
>LPL00187-LactobacillusplantarumWCFS1,LPL_167690_169192
ATGTCAAACGATTGACAT
>PEP01800-PediococcuspentosaceusATCC25745,NC_008525_574186_575688
ATGTCAAACGATTGACAT
>EF00492-EnterococcusfaecalisV583,NC_004668_1557238_1558695
ATGTTATACGTTTGACTT
>SAG01646-Streptococcusagalactiae2603V/R,NC_004116_1691408_1692844
ATGTCAAGCGTTTGACAT
>SPM01882-StreptococcuspyogenesM18MGAS8232,NC_003485_1562572_1564008
ATGTCAAGCGTTTAACAT
>MN00134-StreptococcusmutansUA159,Contig2_1741398_1742834
ATGTCAATCGTTTTACAT
>LSK01793-Lactobacillussakeisubsp.sakei23K,CR936503_1779379_1780851
ATGTCAAACGATTATCAT
>LSL00065-Lactobacillussalivariussubsp.salivariusUCC118(IG-157),NC_007929_81998_80511
ATGTTATACGATTGACAG
>LAC00383-LactobacillusacidophilusNCFM,NC_006814_375108_373654
ATGTCAATCGTTTGACAC
>LJO00518-LactobacillusjohnsoniiNCC533,NC_005362_1861776_1863242
ATGTCAAGCGCTTATCAT
>LGA00093-LactobacillusgasseriATCC-33323(JGI),NC_008530_1764253_1765719
ATGTCAAGCGCTTATCAC
>STH00541-StreptococcusthermophilusCNRZ1066(IG-40),NC_006449_1552093_1553532
ATGTCAAACGTTTAGCAT
2256
>LPL02256-LactobacillusplantarumWCFS1,LPL_2040324_2039317
CATGAAAGCGATTTCAAT
>LBE01171-LactobacillusbrevisATCC367,NC_008497_1178074_1177076
TTCGAAAACGTTTTCAAT
>LSE00755-LactobacilluscaseiATCC334,NC_008526_804301_805299
CAGTAAAACGCTTGCATT
>LSL00420-Lactobacillussalivariussubsp.salivariusUCC118(IG-157),NC_007929_463530_464534
TTATGAAACGCTTGCAAT
>LME00420-LeuconostocmesenteroidesATCC-8293(JGI),scaffold_19_12466_11462
GTTGTAAGCGTTTGCAAT
>LDB00875-Lactobacillusdelbrueckiisubsp.bulgaricusATCC11842(IG-98),NC_008054_1368486_1367488
GCTGTAAGCGATTGCACC
>LJO00491-LactobacillusjohnsoniiNCC533,NC_005362_545423_546415
GTCGTAAACGGTTGCAAT
>LGA00804-LactobacillusgasseriATCC-33323(JGI),NC_008530_462533_463570
GTCGTAAACGGTTGCAAT
>LAC00414-LactobacillusacidophilusNCFM,NC_006814_405409_406407
GCTGTAAACGTTTGCAAT
>PPE01697-PediococcuspentosaceusATCC25745(JGI),scaffold_9_21906_20905
TTTGATAACGTATTCATG
2602
>LPL02602-LactobacillusplantarumWCFS1,LPL_2322719_2321784
CCTGGTACCGGTTTCACA
>SAG00762-Streptococcusagalactiae2603V/R,NC_004116_781580_782386
CATGGAACGGGTTCCAGC
>LSK01554-Lactobacillussakeisubsp.sakei23K,CR936503_1526189_1527010
CCTGGGACGCGTTTCATA
3221
>LPL03221-LactobacillusplantarumWCFS1,LPL_2871483_2870485
TATGGAAACGATTCCAAA
>LSE01604-LactobacilluscaseiATCC334,NC_008526_2095700_2094702
AATGAAATCGATTCCATA
3531
>LPL03533_1-LactobacillusplantarumWCFS1,LPL_3150332_3151750
AGTGTAAACGTTTACTTT
>LPL03533_2-LactobacillusplantarumWCFS1,LPL_3150332_3151750
ATTGTAAACGTTTACGTT
>OON00187_1-OenococcusoeniPSU-1,NC_008528_40575_39163
ACAGTAAACGTTTACTAT
>OON00187_2-OenococcusoeniPSU-1,NC_008528_40575_39163
ATTGTAAACGTTTACTGT
>OON00187_3-OenococcusoeniPSU-1,NC_008528_40575_39163
TTTGTAAACGTTTACTTA
>LRE01125-LactobacillusreuteriJCM1112,AAOV01000033_9204_7525
TAAGTAAACGTTTACATT
>LME01514-LeuconostocmesenteroidesATCC-8293(JGI),scaffold_57_4727_6394
ATTGTAAACGTTTACGCA
3625
>LPL03627-LactobacillusplantarumWCFS1,LPL_3242794_3241121
TAAGCAAACGTTTGCTTA
>LBE02143_1-LactobacillusbrevisATCC367,NC_008497_2145014_2146393
TACACAAACGTTTGCTTT
>LBE02143_2-LactobacillusbrevisATCC367,NC_008497_2145014_2146393
ATTGCAAACGTTTGTGTA
>SHA00075-StaphylococcushaemolyticusJCSC1435,NC_007168_82213_80498
TACTCAAACGTTTGAGTA
3661
>LPL03660-LactobacillusplantarumWCFS1,LPL_3281249_3280332
TCATAAAACGTTTTAATA
>EF00346-EnterococcusfaecalisV583,NC_004668_2834340_2833432
TACTAAAACGTTTTAATT
>PEP00437-PediococcuspentosaceusATCC25745,NC_008525_1674652_1673747
TCATAAAACGTTTTAAAC
>EFA02850-EnterococcusfaeciumDO(JGI),Contig0036_2675_1746
TACTAAAACGTTTTAAAA
>LSK00196-Lactobacillussakeisubsp.sakei23K,CR936503_193092_193973
TAGTAAAACGTTTTACTA
>SAM00277-StaphylococcusaureusMu50,NC_002758_315158_314280
TGGTAAAACGTTTTACTA
3470
>LMS00828
ATCATTTACTAATTTTT
>LMS00828
TAAATTTAGTAATGAAA
>LMS00828
ACATTTGACTAATGTAA
>LPL03469
TTATTTTAGTAAAACGG
>LPL03469
GATATTTAGTAATTATT
3479
>LSE00410
TATTTTTAGTAAAATAA
>LSE00410
AATTTTTAGTAAAAGGA
>LPL03482
AAATTTTAGTAAAACTA
>LPL03482
TCGTTTTAGTTAAACTT
>OOE01095
GGAATTTAGTAAATATT
>LRE01263
TGTATTTAGTAAAAGTT
>LSK00764
AACATTTACTAAAAAAT
>LSK00765
ATTTTTTAGTAAATGTT
>LSK00764
TCATTTTACTAAACATT
>LSK00765
AATGTTTAGTAAAATGA
>LSL00381
AAAGTTTACTAAAAAGT
>LSL00380
ACTTTTTAGTAAACTTT
>LSL00381
TATTTTTAGTAAATTTT
>LSL00380
AAAATTTACTAAAAATA
>LBE01859
AAACTTTACTAAAAATT
>MN01418
AAATTTTACTAAATTTA
>MN00620
TAAATTTAGTAAAATTT
>MN01418
TATTTTTACTAAAATTT
>MN00620
AAATTTTAGTAAAAATA
>PN00747
AAATTTTAGTAAAATAT
>PN00746
ATATTTTACTAAAATTT
>PN00747
CCTTTTTACTAAAATTT
>PN00746
AAATTTTAGTAAAAAGG
>STH00745
ATATTTTACTAAAAAAA
>STH00744
TTTTTTTAGTAAAATAT
3488
>PEP01517
AAATTTTACTAAAAAAC
>PEP01517
CTAATTTACTAAAATAA
>LPL03486
AAATTTTACTAAAAATC
>PEP00412
AATATTTACTCAATCCC
>PEP00412
TATTTTAACTAAAATAT
>LPL03487
AAGTTTTACTCAACTTC
>LPL03487
TCTTTTAACTAAAAGTT
