# Supplementary material for: A generic approach to identify Transcription Factor-specific operator motifs; Inferences for LacI-family mediated regulation in Lactobacillus plantarum WCFS1
Source: BMC Genomics. 2008 Mar 27;9:145. doi: 10.1186/1471-2164-9-145 (PMC2329647; doi:10.1186/1471-2164-9-145)
Supplement: Additional file 9 — The functional annotation of the genes and operons regulated by LacI-TFs in L. plantarum. The file contains a functional description of the genes and operons that putatively constitute the minimal regulons depicted in Figure 3 (with relevant references). [file 1471-2164-9-145-S9.rtf]

The functional annotation of the genes and operons regulated by LacI-TFs in L. plantarum.

The text relates to Figure 3 of the paper: “Identification of LacI-specific operators to link local and global transcription factor activity”.

addendum a)
Lp_0172 (MalR) and Lp_0173 control the transcription of genes related to the uptake and catabolism of maltosaccharides.
The gene lp_0172 is well conserved among the Firmicutes. Knock outs of the ortholog from Streptococcus pneumoniae and Lactococcus lactis, called malR, resulted in constitutive or reduced expression of the regulon for maltose uptake and metabolism, respectively [1, 2]. The difference in effect could be explained by the location of the operator relative to the promoter. Several potential operators were identified in the genome of L. plantarum. The first operator precedes an operon encoding a saccharide/proton symporter (lp_1729), a maltose phosphorylase (lp_1730; map3) and an aldose-1-epimerase (lp_1731; galM2). In fact, only this operator was used to generate the specific motif. Nevertheless, nearly identical Lp_0172 operators were identified upstream of an operon containing an aminotransferase (lp_1721) and an amino acid transporter (lp_1722) and inside a putative operon, upstream of two genes (lp_1818 and lp_1819) related to cell-wall biosynthesis. LacI-TFs have before been implicated in the regulation of genes involved in the transport and catabolism of amino acids in Lactobacilli [3]. Finally, a putative operator was found upstream of the operon encoding the transcription factor itself (lp_0172; malR) and another LacI-TF (lp_0173), and also upstream of the neighboring operon. In addition, the latter region contains an operator for the second LacI-TF, Lp_0173.
Remarkably, almost all Firmicutes species that lack the Lp_0172 (MalR) homolog have an Lp_0173 ortholog and vice versa. Only Leuconostoc mesenteroides and L. plantarum carry both TFs (see Figure 1 of the paper). The Lp_0173 operator was found upsteam of malE which is the first gene of an operon involved in the active transport (ABC transport) and catabolism of malto(oligo)saccharides in L. plantarum (malE, malF, malG, malA, amy2, msmK1, map2). A second operator was found upstream of an operon encoding a transcription anti-terminator (lp_3514; bglG4)(see [4]), which is predicted to regulate the transcription of itself and that of the neighboring operon which includes a PTS system for the uptake of beta-glucosides (lp_3513; pts30BCA) and a 6-phospho-beta-glucosidase (lp_3512; pbg8).

Lp_3221, Lp_3531 and Lp_3625 control the transcription of genes related to the uptake and catabolism of maltosaccharides.
These maltosaccharides probably include short saccharides like sucrose in the case of Lp_3221 and maltose, isomaltose or trehalose in the case of Lp_3531 and Lp3625. Putative operators of Lp_3221 were also found preceding a gene encoding a reductase (lp_1253), a transaldolase (lp_2600) and the EIIA-protein of a sorbitol PTS (lp_2218). The maltosaccharide/proton symporters controlled by Lp_3531 and Lp_3625 (lp_3533 and lp_3626, respectively) are closely related to each other and to the symporter (lp_1729) controlled by Lp_0172 (MalR). The sucrose PTS (lp_3219) controlled by Lp_3221 on the other hand is closely related to the oligofructose/sucrose PTS (lp_0185) controlled by Lp_0188 (SacR).

Lp_0188 (SacR) controls the uptake and catabolism of fructosaccharides.
The related divergently transcribed operons are well conserved among lactobacilli and streptococci and its gene-products were shown to facilitate the regulation, uptake, cleavage and phosphorylation of fructosaccharides in these organisms [5-8]. The regulon in L. plantarum consists of two divergently transcribed operons, one containing lp_0185 (pts1BCA), encoding a oligofructose/sucrose PTS) and lp_0184 (sacK1), encoding a fructokinase, the other containing lp_0187 (sacA or scrB), a beta-fructofuranosidase very similar to a sucrose-6P hydrolase from S. mutans [9], lp_0188 (sacR) and lp_0189 (agl2), which encodes a putative oligofructose-6P hydrolase. Moreover, a putative binding site was found inside the operon containing lp_0172 (malR), lp_0173 and lp_0174, preceding the latter gene. This gene (lp_0174) is orthologous to malA of Staphylococcus xylosus, which encodes a maltase/sucrase with a preference for short saccharides [10].

Lp_3661 (RbsR) controls the uptake and catabolism of ribose.
We identified an operator preceding the ribose utilization operon with which it is associated on the genome. Orthologous operons are found within the genomes of Pediococcus pentosaceus, Enterococcus faecalis and Lactobacillus sakei (see Figure 1 of the paper). The L. sakei operon has been experimentally characterized and the expected regulatory role of the ortholog of Lp_3661 (RbsR) established [11, 12]. The operon includes lp_3660 (rbsK3) (encoding a ribokinase), lp_3659 (rbsD) (encoding a D-ribose mutarotase) and lp_3658 rbsU (encoding a ribose permease). In L. plantarum a second potential operator was found 150 bases upstream of lp_3462 (glpF6), which encodes a glycerol permease. This association agrees well with the need for glycerol-3-P in the conversion of ribose to fructose-6-P [13]. A third potential operator was found upstream of an operon encoding the DNA-helicase RecG and the phospholipid synthesis protein PlsX. Finally, a potential RbsR operator was identified upstream of the operon containing hprK, which encodes a central regulator of carbon catabolism [4].

CcpB.
Putative CcpB operators were found upstream of lp_2069, lp_2503 and lp_2602 (ccpB).  The molecular function of the former gene is unknown. The gene lp_2503 encodes a putative sugar transporter.
 
CcpA.
The motif analysis for CcpA resulted in a good match to the generally accepted operator consensus for this TF. Nevertheless, a search with the specific motif yielded a relatively small number of hits. Considering the fact that CcpA is a global-regulator, it is clear that the CcpA-regulon should be much larger. A good hit was found upstream of ccpA. This upstream regulatory element was experimentally verified for Lactobacillus plantarum WCFS1 and Lactobacillus pentosus [Muscariello, #2001; Mahr, #2000]. Other potential CcpA operators were identified upstream of genes and operons encoding proteins involved in the uptake and catabolism of related carbohydrates. These proteins include a putative N-acetylglucosamine PTS (Lp_2969) a putative glucosamine-6P isomerase (Lp_0226), a phosphoketolase (Lp_3551), a glycerol kinase, glycerol-3P dehydrogenase and a glycerol facilitator (Lp_0370-Lp_0372), a sugar transporter and a sugar kinase (Lp_2594, Lp_2595), a ribokinase and a ribose transporter (Lp_3660-Lp_3658), and a sugar PTS (gene in operon with two hypotheticals). Furthermore, CcpA also appears to be involved in the control of proteolysis.   

addendum b)
Lp_3470 (LacR), Lp_3479 (GalR) and Lp_3488 (RafR) control the transcription of genes related to the uptake and catabolism of lactose, galactose and raffinose/melibiose/galactose, respectively.
A single symmetrical Lp_3470 (LacR) operator was found in between lp_3470 (lacR) and the operon containing lp_3469 (lacS) and lp_3468 (lacA), which encode a PTS-controlled lactose/proton symporter (similar to LacS of Streptococcus thermophilus [14]) and a beta-galactosidase that converts lactose into glucose and galactose (similar to B. subtilis LacA [15]), respectively.
An Lp_3479 (GalR) operator was found upstream of the operon including lp_3482 (galK) (encoding a galactokinase), lp_3481 (galE4) (encoding an UDP_glucose 4_epimerase), lp_3480 (galT) (encoding a galactose-1-phosphate uridyltransferase) and lp_3479 (galR). This operon is orthologous to the one characterized in S. thermophilus [16].
The third regulator, Lp_3488 (RafR), is predicted to be involved in the regulation of lp_3487 (galM3), encoding an aldose-1-epimerase (orthologous to GalM of S. thermophilus [17]), lp_3486 (rafP), encoding a PTS-controlled proton symporter that transports raffinose, melibiose and galactose, and lp_3486 (melA), which encodes an alpha-galactosidase/melibiase. The latter two proteins have been characterized [18]. It was observed that the expression of these genes was not induced by lactose but was induced by raffinose and melibiose. The operators related to Lp_3479 (GalR) and Lp_3488 (RafR) are rather similar, resulting in some cases in overlap in the prediction.

References

1.	Puyet A, Ibáñez AM, Espinosa M: Characterization of the Streptococcus pneumoniae maltosaccharide regulator MalR, a member of the LacI-GalR family of repressors displaying distinctive genetic features. J Biol Chem 1993, 268(34):25402-25408.
2.	Andersson U, Rådström P: Physiological function of the maltose operon regulator, MalR, in Lactococcus lactis. BMC Microbiol 2002, 2:28.
3.	Schick J, Weber B, Klein JR, Henrich B: PepR1, a CcpA-like transcription regulator of Lactobacillus delbrueckii subsp. lactis. Microbiology 1999, 145:3147-3154.
4.	Deutscher J, Francke C, Postma PW: How phosphotransferase system-related protein phosphorylation regulates carbohydrate metabolism in bacteria. Microbiol Mol Biol Rev 2006, 70(4):939-1031.
5.	Hiratsuka K, Wang B, Sato Y, Kuramitsu H: Regulation of sucrose-6-phosphate hydrolase activity in Streptococcus mutans: characterization of the scrR gene. Infect Immun 1998, 66(8):3736-3743.
6.	Luesink EJ, Marugg JD, Kuipers OP, de Vos WM: Characterization of the divergent sacBK and sacAR operons, involved in sucrose utilization by Lactococcus lactis. J Bacteriol 1999, 181(6):1924-1926.
7.	Barrangou R, Altermann E, Hutkins R, Cano R, Klaenhammer TR: Functional and comparative genomic analyses of an operon involved in fructooligosaccharide utilization by Lactobacillus acidophilus. Proc Natl Acad Sci USA 2003, 100(15):8957-8962.
8.	Saulnier DM, Molenaar D, de Vos WM, Gibson GR, Kolida S: Identification of prebiotic fructooligosaccharide metabolism in Lactobacillus plantarum WCFS1 through microarrays. Appl Environ Microbiol 2007, 73(6):1753-1765.
9.	Lunsford RD, Macrina FL: Molecular cloning and characterization of scrB, the structural gene for the Streptococcus mutans phosphoenolpyruvate-dependent sucrose phosphotransferase system sucrose-6-phosphate hydrolase. J Bacteriol 1986, 166(2):426-434.
10.	Egeter O, Brückner R: Characterization of a genetic locus essential for maltose-maltotriose utilization in Staphylococcus xylosus. J Bacteriol 1995, 177(9):2408-2415.
11.	Stentz R, Zagorec M: Ribose utilization in Lactobacillus sakei: analysis of the regulation of the rbs operon and putative involvement of a new transporter. J Mol Microbiol Biotechnol 1999, 1(1):165-173.
12.	Stentz R, Cornet M, Chaillou S, Zagorec M: Adaptation of Lactobacillus sakei to meat: a new regulatory mechanism of ribose utilization? Lait 2001, 81:131-138.
13.	Kim I, Kim E, Yoo S, Shin D, Min B, Song J, Park C: Ribose utilization with an excess of mutarotase causes cell death due to accumulation of methylglyoxal. J Bacteriol 2004, 186(21):7229-7235.
14.	Poolman B, Royer TJ, Mainzer SE, Schmidt BF: Lactose transport system of Streptococcus thermophilus: a hybrid protein with homology to the melibiose carrier and enzyme III of phosphoenolpyruvate-dependent phosphotransferase systems. J Bacteriol 1989, 171(1):244-253.
15.	Daniel RA, Haiech J, Denizot F, Errington J: Isolation and characterization of the lacA gene encoding beta-galactosidase in Bacillus subtilis and a regulator gene, lacR. J Bacteriol 1997, 179(17):5636-5638.
16.	Vaillancourt K, Moineau S, Frenette M, Lessard C, Vadeboncoeur C: Galactose and lactose genes from the galactose-positive bacterium Streptococcus salivarius and the phylogenetically related galactose-negative bacterium Streptococcus thermophilus: organization, sequence, transcription, and activity of the gal gene products. J Bacteriol 2002, 184(3):785-793.
17.	Poolman B, Royer TJ, Mainzer SE, Schmidt BF: Carbohydrate utilization in Streptococcus thermophilus: characterization of the genes for aldose 1-epimerase (mutarotase) and UDPglucose 4-epimerase. J Bacteriol 1990, 172(7):4037-4047.
18.	Silvestroni A, Connes C, Sesma F, Savoy de Giori G, Piard JC: Characterization of the melA locus for alpha-galactosidase in Lactobacillus plantarum. Appl Environ Microbiol 2002, 68(11):5464-5471.
